# Supplementary figures and images for: Efficacy and safety of tranexamic acid on blood loss and seizures in patients undergoing meningioma resection: A systematic review and meta-analysis
Source: PLoS One. 2024 Sep 4;19(9):e0308070. doi: 10.1371/journal.pone.0308070 (PMC11373793; doi:10.1371/journal.pone.0308070)

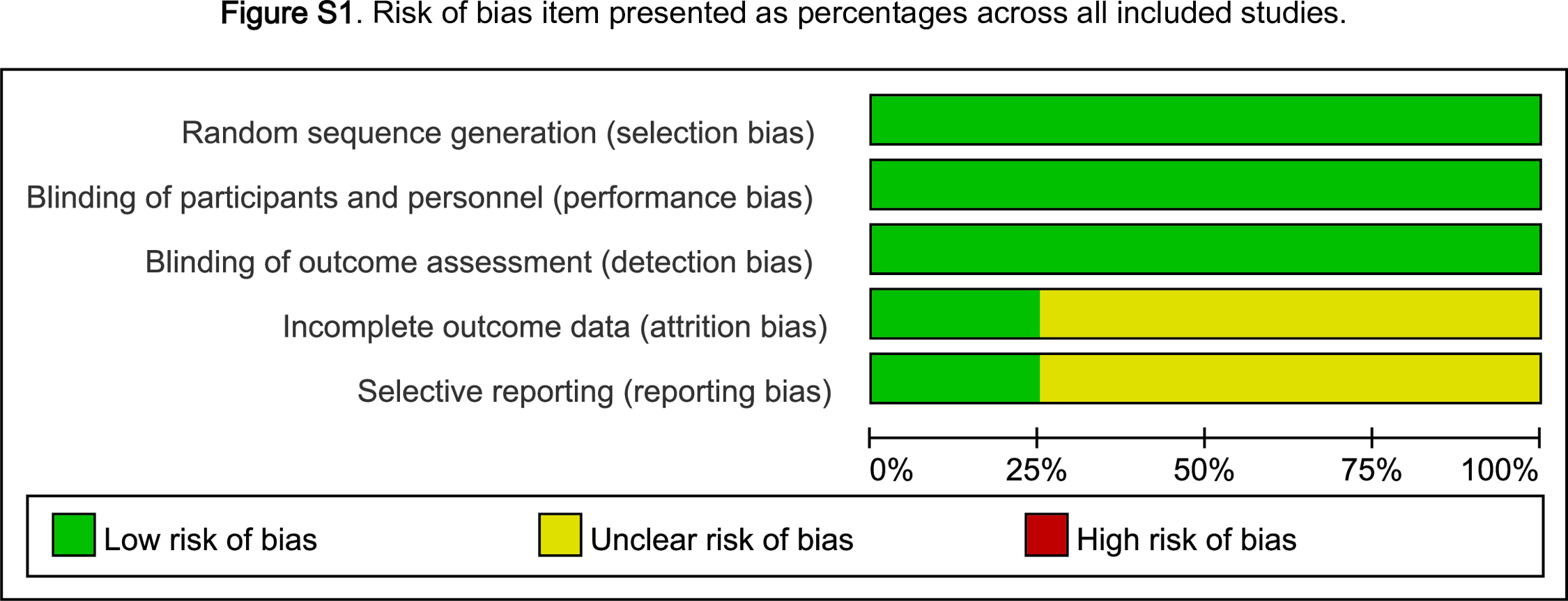

Supplement: S1 Fig — (TIF) [file pone.0308070.s002.tif]

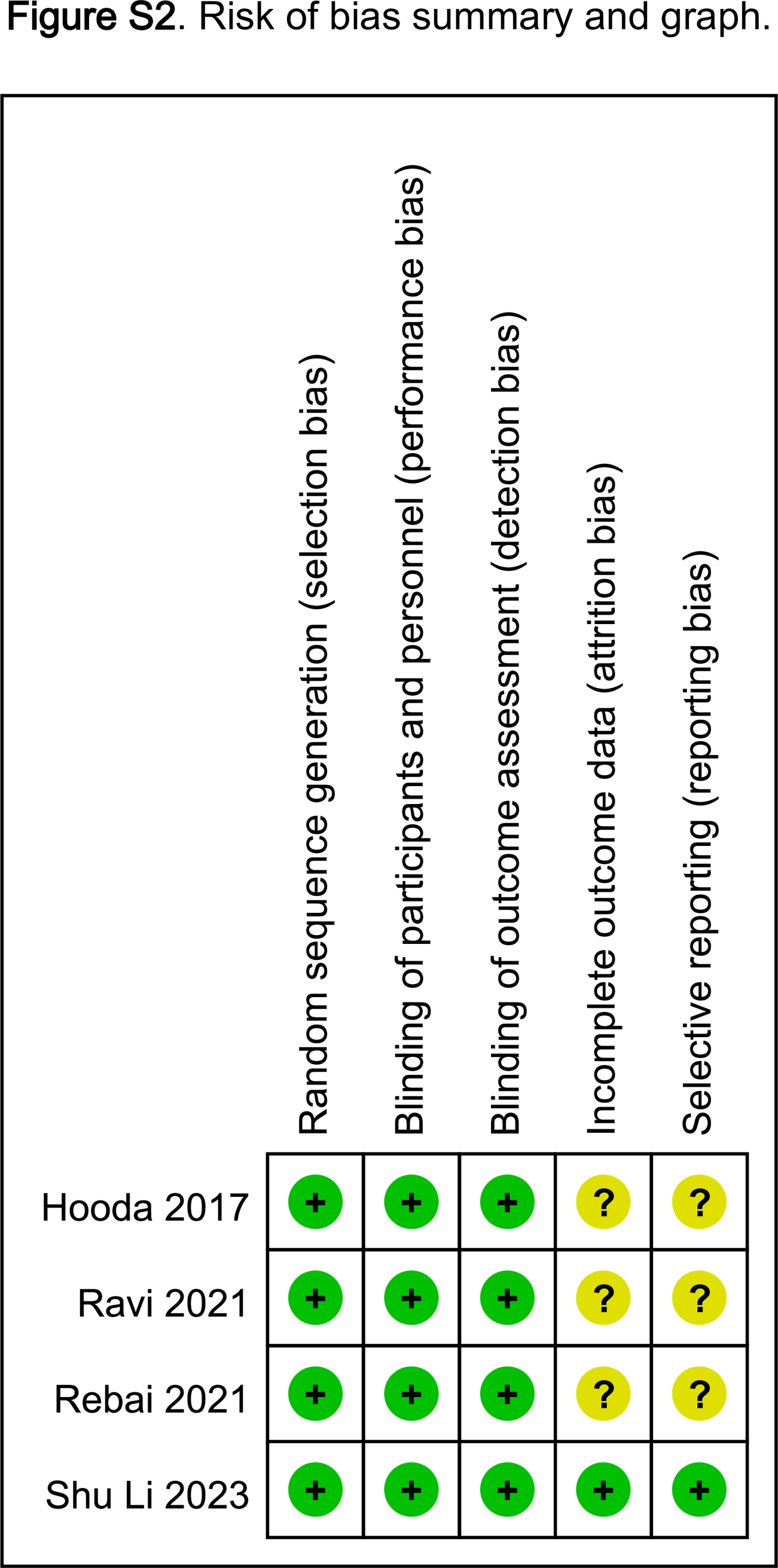

Supplement: S2 Fig — (TIF) [file pone.0308070.s003.tif]
