# Supplementary material for: Efficacy and safety of tranexamic acid on blood loss and seizures in patients undergoing meningioma resection: A systematic review and meta-analysis
Source: PLoS One. 2024 Sep 4;19(9):e0308070. doi: 10.1371/journal.pone.0308070 (PMC11373793; doi:10.1371/journal.pone.0308070)
Supplement: S1 File — (ZIP) [file pone.0308070.s004.zip › 2017_JCN_Hooda_TXA_meningioma_RCT.pdf]

Journal of Clinical Neuroscience xxx (2017) xxx–xxx

Contents lists available at ScienceDirect

Journal of Clinical Neuroscience

journal homepage: www.elsevier.com/locate/jocn

Case study

Effect of tranexamic acid on intraoperative blood loss and transfusion requirements in patients undergoing excision of intracranial meningioma

Bhavna Hooda [a], Rajendra Singh Chouhan [a], Girija Prasad Rath [a,*], Parmod Kumar Bithal [a], Ashish Suri [b], Ritesh Lamsal [a]

[a] *Department of Neuroanaesthesiology & Critical Care, All India Institute of Medical Sciences (AIIMS), New Delhi, India*
[b] *Department of Neurosurgery, All India Institute of Medical Sciences (AIIMS), New Delhi, India*

ARTICLE INFO

Article history:
Received 20 December 2016
Accepted 13 February 2017
Available online xxxx

Presented at the 50th Anniversary Annual Scientific Meeting of Neuroanaesthesia Society of Great Britain & Ireland held at Manchester, UK on 7–8 May 2015

Keywords:
Tranexamic acid
Neurosurgery
Meningioma
Intraoperative blood loss
Blood transfusion

ABSTRACT

Surgical excision of meningioma is often complicated by significant blood loss requiring blood transfusion with its attendant risks. Although tranexamic acid is used to reduce perioperative blood loss, its blood conservation effect is uncertain in neurosurgery. Sixty adults undergoing elective craniotomy for meningioma excision were randomized to receive either tranexamic acid or placebo, initiated prior to skin incision. Patients in the tranexamic acid group received intravenous bolus of 20 mg/kg over 20 min followed by an infusion of 1 mg/kg/h till the conclusion of surgery. Intraoperative blood loss, transfusion requirements and estimation of surgical hemostasis using a 5-grade scale were noted. Postoperatively, the extent of tumor excision on CT scan and complications were observed. Demographics, tumor characteristics, amount of fluid infusion, and duration of surgery and anesthesia were comparable between the two groups. The amount of blood loss was significantly less in tranexamic acid group compared to placebo (830 ml vs 1124 ml; $p = 0.03$). The transfusion requirement was less in tranexamic acid group ($p > 0.05$). The patients in tranexamic acid group fared better on a 5-grade surgical hemostasis scale with more patients showing good hemostasis ($p = 0.007$). There were no significant differences between the groups with regards to extent of tumor removal, perioperative complications, hospital stay or neurologic outcome. To conclude, administration of tranexamic acid significantly reduced blood loss in patients undergoing excision of meningioma. Fewer patients in the tranexamic acid group received blood transfusions. Surgical field hemostasis was better achieved in patients who received tranexamic acid.

© 2017 Elsevier Ltd. All rights reserved.

1. Introduction

Meningiomas account for almost 30% of primary brain tumors. Although, 80–90% of these are benign [1], surgical excision of meningiomas are often complicated by significant blood loss. Major intra-operative blood loss may lead to life-threatening hemodynamic instability requiring massive transfusion of crystalloids, colloids and allogeneic blood. Allogenic blood transfusion has its attendant risks of transmitted infections, post-operative sepsis, immune modulation and an undue wastage of a scarce resource [2,3]. Therefore, there is an increasing focus on strategies to minimize surgical blood loss by employing modalities like preoperative erythropoietin, autologous pre-donation, peri-operative blood salvage and use of pharmacologic agents such as fibrin sealants, antifibrinolytics, desmopressin and recombinant Factor VII [4].

Anti-fibrinolytics are commonly used pharmacological agents in modern blood conservation strategy. Tranexamic acid is a synthetic lysine analog (trans-4-aminomethyl-cyclohexane-1-carboxylic acid) that acts as a competitive inhibitor of plasmin and plasminogen, preventing clot dissolution [5,6]. Its potential to reduce perioperative blood loss has been proven in a variety of surgical procedures [7–12]. However, in the field of neurosurgery, its use is mostly limited to spinal and extracranial surgery [13]. Although tranexamic acid was evaluated way back in the late 1990s for preventing re-bleeding in subarachnoid hemorrhage [14,15], there is a renewed interest now in the same area.

Therefore, this prospective, randomized, double blind, placebo controlled study was conducted to evaluate the effect of tranexamic acid on intra-operative blood loss and transfusion requirements in patients undergoing elective craniotomy for intracranial

* Corresponding author at: Department of Neuroanaesthesiology & Critical Care, Neurosciences Centre, A.I.I.M.S., New Delhi 110029, India. Fax: +91 11 26588663.
E-mail address: girijarath@yahoo.co.in (G.P. Rath).

http://dx.doi.org/10.1016/j.jocn.2017.02.053
0967-5868/© 2017 Elsevier Ltd. All rights reserved.

Please cite this article in press as: Hooda B et al. Effect of tranexamic acid on intraoperative blood loss and transfusion requirements in patients undergoing excision of intracranial meningioma. J Clin Neurosci (2017), http://dx.doi.org/10.1016/j.jocn.2017.02.053

meningiomas. As secondary outcome measures, the effect of tranexamic acid on the quality of surgical hemostasis, perioperative complications, length of hospital stay and neurological outcome were also evaluated.

2. Materials and methods

2.1. Study population

When this project was conceived, literature search did not reveal any study on intraoperative use of tranexamic acid in intracranial procedures. Hence, we extrapolated data from tranexamic acid efficacy in spine surgery [13] and assumed a blood loss reduction of 25% that would entail a sample size of 60 to give the study a power of 80% and alpha error of 0.5.

2.2. Methodology

After obtaining approval from the Institutional Ethics Committee, 60 consecutive American Society of Anesthesiologists (ASA) grade I and II patients, aged between 18 and 60 years, of either sex, scheduled to undergo excision of intracranial meningioma were enrolled in the study, after obtaining a written informed consent. All patients were operated upon by a neurosurgeon with at least 3 years of experience.

Patients who refused to participate in the study or were allergic to tranexamic acid, had a history suggestive of bleeding diathesis, thromboembolic episode prior to surgery or family history of thromboembolism, patients on medication that could interfere with coagulation, epilepsy, plasma creatinine values more than 1.5 mg/dl and pregnant or lactating mothers were excluded from the study. Patients who were planned for preoperative embolization, with tumor size less than 4 cm or operating neurosurgeon's estimate of likely intra-operative blood loss less than 20% of patient's estimated blood volume (EBV) were also not enrolled.

2.3. Anesthetic technique and intraoperative management

In the operation theatre, after connecting non-invasive monitors, intravenous (IV) access was secured and left radial artery was cannulated following local infiltration of 2% lignocaine. A baseline blood sample was analyzed to rule out any pre-existing coagulopathy or hyperfibrinolysis. Corrective measures was planned if the findings were clinically relevant and confirmed on standard laboratory hemostatic tests [SLTs as Prothombin time (PT), activated partial thromboplastin time (aPTT), International normalized ratio (INR) and Platelet count].

After recording the baseline vital parameters, induction of anesthesia was done with propofol (2 mg/kg) preceded by fentanyl (2 μg/kg). Rocuronium (1 mg/kg) was administered to facilitate tracheal intubation. Anesthesia was maintained with 60% nitrous oxide in oxygen mixture, sevoflurane (1–1.5 MAC) and supplemental boluses of rocuronium and fentanyl. Mechanical ventilation was adjusted to maintain an end-tidal carbon-dioxide ($EtCO_2$) of 30–35 mmHg with a fresh gas flow of 2 L/min. Throughout the surgery, HR and MAP were maintained within 20% of the baseline and any deviations managed as per standard practice. Normothermia (36–37 °C) was maintained with the help of convective air warmers and warm IV fluid administration.

2.4. Group allocation

The patients were randomized to receive either tranexamic acid (Group T) or normal saline (Group P, Placebo) based on a computer-generated randomization chart (See Fig. 1).

2.5. Preparation and administration of "test drug" infusion

The study drugs were prepared by an anesthesiologist who was part of the study but not involved in the patient management. For Group T patients, 2000 mg of tranexamic acid was diluted to 50 ml with normal saline (40 mg/ml) in a 50 ml syringe and 50 ml normal saline was taken in same sized syringe for Group P patients. In both the groups, the syringes were labeled as "Test drug" for blinding of the attending anesthesiologists.

The test drug was administered (0.5 ml/kg) over 20 min (tranexamic acid 20 mg/kg) as a loading dose before skin incision followed by a maintenance infusion of 0.025 ml/kg/h (tranexamic acid 1 mg/kg/h) till the completion of skin suture. Infusion of 20% mannitol (1 gm/kg) was completed before dural opening. Hemodynamic variables, $EtCO_2$, oxygen saturation, ST-segment analysis, peak airway pressure, nasopharyngeal temperature and input/ output were continuously monitored. Blood loss estimation in all the cases was done by the principal investigator by subtracting the amount of irrigation fluid from suction aspirate and visual assessment of the soaked sponges, cotton pledgets and area at the operating end. The principle investigator as well as the surgeon were blinded to the test drug. A note of all intra-operative complications was made. Intravenous fluid administration consisted of isotonic crystalloids and colloids (tetrastarch). Transfusion of blood/blood products and intra-operative blood salvage was made at the discretion of the attending anesthesiologist. The hemostatic therapy was guided by ASA task force on blood transfusion. After the maximum possible tumor excision had been achieved, surgeon's estimate of oozing/hemostasis was recorded based on fixed parameters (Appendix I) [18]. At the completion of the skin suture, sevoflurane, nitrous oxide and infusion of the "Test drug" were discontinued. After satisfactory reversal of residual neuromuscular blockade, trachea was extubated. When indicated, elective post-operative ventilation was continued and reasons for ventilation was recorded.

2.6. Postoperative care

All the patients were monitored post-operatively in the neurosurgical ICU. Perioperative, transfusion trigger for packed RBCs was a hemoglobin concentration <8 g/dl. Fresh frozen plasma (FFP) was transfused at INR > 1.5 and for transfusing platelets, a platelet count <100,000/mm^3 was considered as the cut-off value [19]. The amount of transfused packed RBCs, FFP and / or platelets were recorded.

In addition to routine laboratory investigations like hemoglobin concentration, SLT's, blood urea nitrogen, creatinine, electrolytes, chest X-ray and ECG were performed on the first post-operative day. Computed tomography (CT) scan findings for the extent of tumor removal (Simpson's grade) [20] and hematoma formation were recorded. Any complications in the post-operative period were recorded with special emphasis on thrombotic events i.e. stroke, deep venous thrombosis (DVT), pulmonary embolism (PE), myocardial ischemia/ infarction, acute renal failure, new onset seizures and visual abnormalities. The diagnosis of myocardial infarction was based on the appearance of significant new ST-segment changes, confirmed by Troponin I test. Ischemic stroke was defined as a focal neurologic deficit lasting more than 24 h, confirmed by a non-contrast CT scan brain and the opinion of attending neurosurgeon. A decrease in urine output below 0.5 ml/kg/h for more than six hours and an increase in post-operative serum creatinine by 1.5-fold was required for the diagnosis of acute renal failure. If DVT/PE was suspected on clinical grounds and laboratory parameters (D-dimer), additional tests like extremity ultrasound, chest X-ray and/or CT scan were performed. The duration of ICU and hospital stay were recorded. Patients' physical status at the time of discharge from the hospital was

Please cite this article in press as: Hooda B et al. Effect of tranexamic acid on intraoperative blood loss and transfusion requirements in patients undergoing excision of intracranial meningioma. J Clin Neurosci (2017), http://dx.doi.org/10.1016/j.jocn.2017.02.053

graded as per the Extended Glasgow outcome scale (GOSE) [21] and was categorized as good recovery (GOSE 7–8), moderate disability (GOSE 5–6), and severe disability (GOSE 1–4).

2.7. Statistical analysis

Statistical analysis was performed with STATA 12.1 (College Statistics, Texas, USA). Continuous variables like age, weight, laboratory parameters, baseline temperature, intravenous crystalloids, duration of surgery and anesthesia are expressed as mean ± SD and non-parametric variables like tumor size, midline shift, colloids and duration of mechanical ventilation are expressed as median (range). Demographics, laboratory values, tumor location, extent of tumor removal, surgical grade of ooze, amount of intraoperative crystalloids infused, use of cell saver, duration of surgery, post-operative complications and length of hospital stay were compared between the groups by using the two sample Student's t test for mean values and Pearson χ^2 test or Fisher's exact test for proportions. Willcoxon rank-sum (Mann–Whitney) test was used to compare nonparametric variables like tumor size, amount of midline shift, colloid use, amount of autologous blood infused intra-operatively, amount of post-operative blood transfusion, duration of mechanical ventilation and length of ICU stay. Shapiro–Wilk test was performed to test for normality of distribution of continuous variables. Blood loss and intra-operative transfusion requirements did not follow Gaussian distribution; therefore these variables were analyzed after log transformation and expressed as geometric mean (difference). A p value < 0.05 indicated statistical significance.

3. Results

Sixty patients (30 in each group) were recruited on the basis of inclusion and exclusion criteria laid down in the methodology (Fig. 1). Patients underwent craniotomy for excision of intracranial meningioma located at cerebral convexity (17), infratentorial (17), parasaggital (12), skull base (09) and juxtasellar (05) regions. The two groups were comparable in terms of demographics, preoperative laboratory parameters and tumor characteristics (Table 1). Amount of fluids, duration of surgery and anesthesia were also comparable in the two groups (Table 2). The average dose of tranexamic acid received in group T was 1612 mg. The hemodynamic parameters (HR and MAP) remained stable throughout the intra-operative period, in both groups.

The amount of blood loss was significantly less in the tranexamic acid group compared to placebo group (Table 3 and Fig. 2). The trend was towards lesser transfusion requirement in the tranexamic acid group; even though it was not statistically significant. Number of patients receiving autologous transfusion and its amount were comparable between the two groups (Table 4).

The extent of tumor removal was similar between groups (Table 5). Complete excision of tumor (Simpson's grade I and II excision) was achieved in 50 (83.3% of total) patients, 80% of patients in group T and 86% of patients in group P. Patients in Group T fared better on a 5-grade surgical hemostasis scale with more patients showing good hemostasis (grade 0–1) as compared to placebo. Occurrence of hematoma was also comparable in both the groups.

Postoperatively, hemoglobin and coagulation profile did not show statistical difference between the groups. Incidence of postoperative complications was comparable in the groups (Table 6). One patient in Group T and three (10%) in group P needed additional surgical intervention. One patient required decompressive craniectomy because of malignant venous infarct and evacuation of pin-site EDH, whereas two patients were re-explored for swollen residual tumor. Postoperative blood transfusion over 24 h was analyzed between groups and was comparable (p = 0.08).

The patient outcome was not affected by intraoperative use of tranexamic acid (Table 6). One patient in Group T died due to acute respiratory distress syndrome (ARDS) subsequent to aspiration on the 16th postoperative day. Duration of ICU and hospital stay were also similar in the two groups.

4. Discussion

In this study, tranexamic acid significantly reduced the amount of blood loss in adults undergoing elective craniotomy for intracranial meningioma (p = 0.03). There was a mean reduction of blood loss of 300 ml with the use of tranexamic acid as compared to placebo. The number of patients requiring transfusion was also lower (n = 13) compared to placebo (n = 17). In addition, after tumor excision, hemostasis was significantly better in tranexamic acid group. Though, the difference in the amount of blood transfusion did not show statistical significance, there was a trend towards lesser amount of transfused PRBC in tranexamic acid group (554 ml vs. 645 ml) compared to the placebo group. Vel R and colleagues [16] also found reduced blood loss with the use of tranexamic acid which did not translate into reduced blood transfusion.

Traditionally, excision of intracranial meningioma is known to result in major blood loss requiring large volume of allogeneic transfusions of blood and blood products. This is attributed to the unique blood supply of meningiomas from meningeal vessels and parasitation of pial vasculature, difficult vascular supply of skull base meningiomas, obscured surgical plane in large tumors with significant peritumoral edema; major cerebral artery encasement by the meningioma, tumor involvement of dural venous sinuses and invasion of scalp and calvaria with associated feeders [22]. There is some suggestion that leptomeninges may be rich in tissue plasminogen activator; therefore it may be hypothesized that blood loss in intracranial meningiomas could be aggravated by local tissue plasminogen activator (t-PA) induced hyperfibrinolysis which adds on to the consumptive coagulopathy induced by the surgical stress and dilutional coagulopathy that is known to accompany protracted intracranial surgery [23,24].

Reducing perioperative bleeding with administration of antifibrinolytics has been proven in diverse surgical settings [7–12]. However, in the context of intracranial surgeries, majority of literature revolves around antifibrinolytic use in reducing aneurysmal rebleed [15], and the blood sparing potential of tranexamic acid during intracranial tumor excision remains an unexplored domain. In the present study, we could demonstrate a significant reduction in intraoperative blood loss by almost a quarter with the use of tranexamic acid during excision of meningiomas which is similar to the study carried out by Vel et al. [16] Tranexamic acid treated group fared favorably in terms of hemostasis on a surgeon-graded subjective oozing scale. By reducing the magnitude of blood loss, tranexamic acid might allow more complete tumor excision. However, in the present study, it did not affect the extent of tumor removal with Simpson's grade I and II excision achieved in almost equal number of patients in the two groups. As surgical excision largely depends on tumor location (supratentorial/ skull base/ infratentorial); intuitively, tranexamic acid was unlikely to play a role in the extent of tumor removal.

A particular neurosurgical concern with meningiomas is a high rate (6–8%) of postoperative hematoma that typically presents within 6 h [25]. In this context, a noteworthy pharmacokinetic profile of this drug is the terminal elimination half-life of 12 h and therefore extending this hemostatic effect into the crucial postoperative period and reducing the need for re-exploration for intracranial hematoma [5,6]. The CRASH-2 (Clinical Randomisation

Please cite this article in press as: Hooda B et al. Effect of tranexamic acid on intraoperative blood loss and transfusion requirements in patients undergoing excision of intracranial meningioma. J Clin Neurosci (2017), http://dx.doi.org/10.1016/j.jocn.2017.02.053

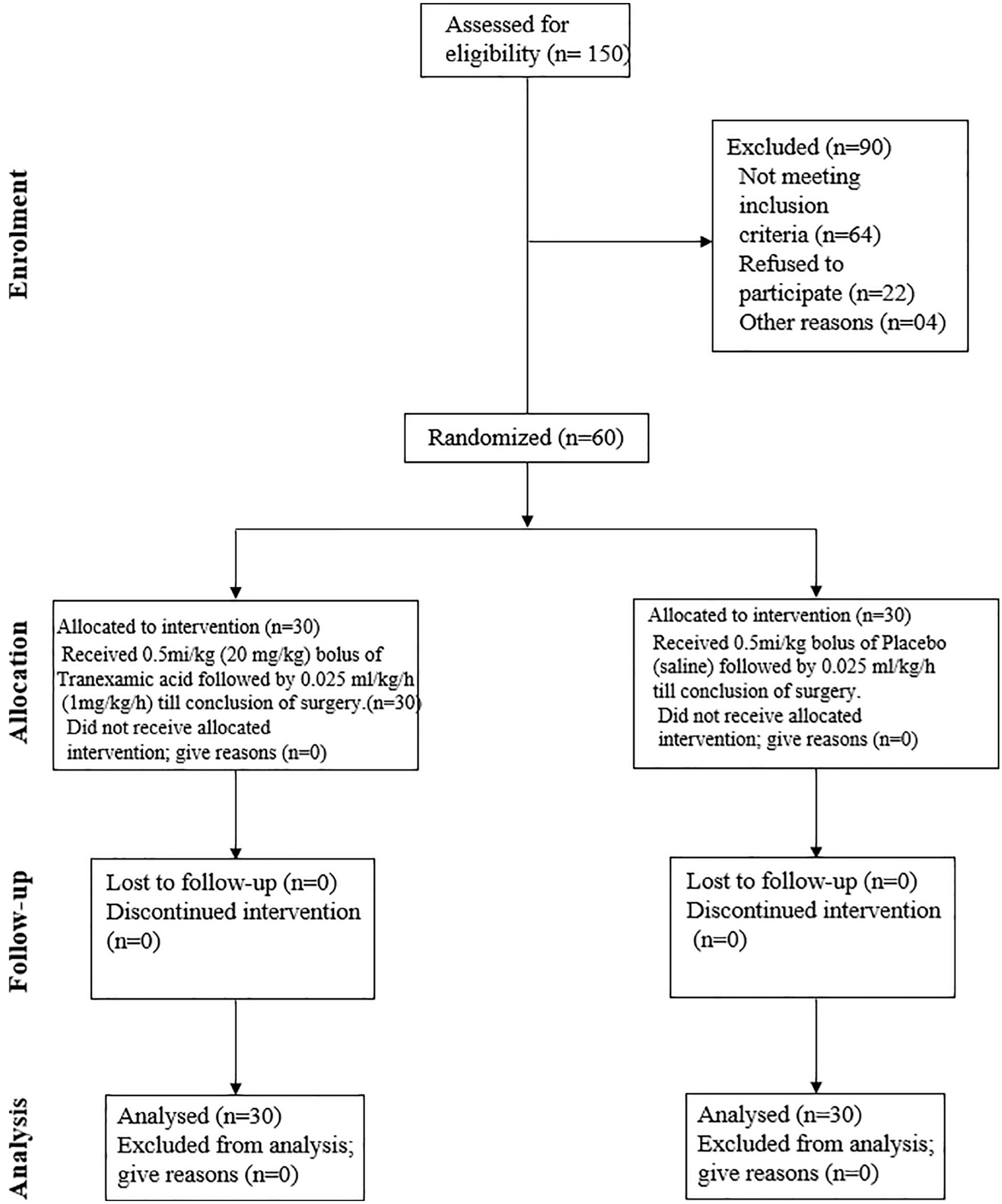

Fig. 1. Consort diagram of the study.

of an Antifibrinolytic in Significant Hemorrhage) trial evaluated the effect of tranexamic acid in 270 patients with traumatic brain injury and found less bleeding progression and mortality in the treated group in comparison to patients who received placebo [26,27]. However, in our study, SDH/EDH/operative cavity bleed developed in four patients in placebo group compared to three patients in tranexamic acid group. This finding did not reach statistical significance due to small patient population. As post-operative hemorrhage can have a devastating outcome in neurosurgical patients, this necessitates further studies with larger

Please cite this article in press as: Hooda B et al. Effect of tranexamic acid on intraoperative blood loss and transfusion requirements in patients undergoing excision of intracranial meningioma. J Clin Neurosci (2017), http://dx.doi.org/10.1016/j.jocn.2017.02.053

ARTICLE IN PRESS

Table 1
Demographic profile, preoperative laboratory parameters and tumor characteristics.†

Parameter	Group T ($n = 30$)	Group P ($n = 30$)
Age (years)*	39.3 ± 11.4	41.6 ± 11.2
Male	14 (46.67)	7 (23.3)
Female	16 (53.3)	23 (76.7)
Weight (kg)*	63.7 ± 12.4	58.4 ± 12.9
I	18 (60)	20 (66.7)
II	12 (40)	10 (33.3)
Hemoglobin (g/dl)*	13.0 ± 1.9	12.7 ± 1.7
Platelet count (1×10^3)*	2.2 ± 0.7	1.96 ± 0.65
INR*	1.1 ± 0.1	1.15 ± 0.24
BUN (mg/dl)*	24.6 ± 6.8	24.4 ± 7.1
Creatinine (mg/dl)*	0.8 ± 0.2	0.8 ± 0.2
Tumor location		
Convexity	9 (30)	8 (26.7)
Parasaggital	5 (16.7)	7 (23.3)
Skull base	4 (13.3)	5 (16.7)
Juxta-sellar	2 (6.7)	3 (10)
Infratentorial	10 (33.3)	7 (13.3)
Tumor size (mm^3)$	99 (33, 387)	79 (25, 960)
Midline shift (mm)$	4.5 (0,19)	2 (0,8)

† Data given as number (%) of patients unless specified.
* Data are given as Mean (±SD). ASA: American Society of Anesthesiologists; INR: International normalized ratio; BUN: Blood urea nitrogen.
$ Data given as median (Min, Max).

Table 2
Comparison of intraoperative variables in the two groups.†

Parameter	Group T ($n = 30$)	Group P ($n = 30$)
Baseline HR (beats/min)	88.9 ± 18.6	85.5 ± 17.5
Baseline MAP (mm Hg)	97 ± 14	101 ± 13
Baseline temperature (°C)	36.2 ± 0.5	36.1 ± 0.6
Intra-operative use of cell saver*	7 (23.3)	6 (20)
Crystalloids (ml)	4575	4758
Colloids (ml)‡	1000 (0, 2500)	750 (0, 2500)
Duration of surgery (min)	346 ± 124	359 ± 120
Duration of anesthesia (min)	436 ± 122	447 ± 125

† Data given as Mean ± SD unless specified.
* Data given as number (%) of patients.
‡ Data given as median (Min, Max). HR: Heart rate; MAP: Mean arterial pressure.

cohort of neurosurgical patients to conclusively provide evidence in this direction.

Another noteworthy area is perioperative safety of antifibrinolytics which is under the scanner since FDA suspension of aprotinin [28]. Similar doubts have been casted over safety of tranexamic acid with reported adverse effects such as cerebral ischemia in subarachnoid hemorrhage (SAH), renal dysfunction and active thromboembolic episodes (deep vein thrombosis, pulmonary embolism, myocardial infarction) [29–31]. Another neurologically perturbing implication of high dose tranexamic acid is with non-ischemic seizures in cardiac surgical population [32]. The probable mechanism is the structural homology of tranexamic acid with gamma-amino butyric acid (GABA); thereby, competitively inhibiting inhibitory GABAergic sites in the central nervous system. In the present study, one patient in Group T and two patients in Group P had seizures on first post-operative day. As the study population is too small and was not sufficiently powered to study the complication rate, it is very difficult to make a plausible comment on its association with tranexamic acid. Further, the doses of tranexamic acid used in the study was much lower compared to the reported studies.

There were certain limitations in our study. Reduced blood loss did not translate into statistically significant reduction in amount of blood transfusion. Though fewer patients in the tranexamic acid group received transfusion and findings trended towards a reduced amount of transfused PRBC, it was not statistically significant, possibly due to small effect size (only 50% of the study cohort received transfusion). Secondly, subjective estimation of blood loss by the attending anesthesiologist could have resulted in underestimation of blood lost onto surgical gown, drapes and pledgets. Finally, the

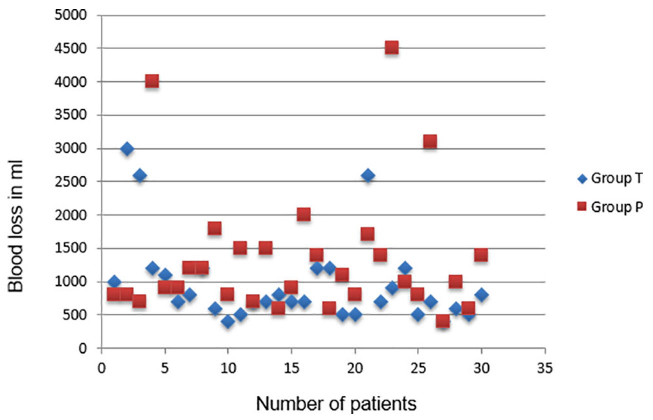

Fig. 2. Estimated blood loss in the two groups of patients.

Table 4
Comparison of intraoperative blood product transfusion.*

Parameter	Group T ($n = 30$)	Group P ($n = 30$)	p value
Cell saver blood			
No of patients transfused	7 (23.3)	6 (20)	
Volume (ml)‡	340 (100, 875)	290 (134, 1410)	0.89
FFP			
No of patients transfused	04 (13.3)	04 (13.3)	
Volume (ml)†	600	900	0.21
PC			
No of patients transfused	03 (10.0)	02 (6.6)	
Volume (ml)†	250	306	0.97

* Data given as number (%) of patients unless specified.
† Data given as mean ± SD.
‡ Data given as median (Min, Max). FFP: fresh frozen plasma; PC: platelet concentrate.

Table 3
Comparison of intra-operative blood loss and transfusion requirements.†

Parameter	Group T ($n = 30$)	Group P ($n = 30$)	Difference (95% CI)	p value
Blood loss (ml)	830	1124	1.3 (1.1,1.8)	0.03
Intra-operative PRBC transfusion				
No of patients transfused*	13 (43.3)	17 (56.7)		
Volume (ml)	554 (382, 803)	645 (498, 834)	1 (0.8, 1.8)	0.46

† Data given as geometric mean (Min, max) of patients unless specified.
* Data given as number (%). PRBC: packed red blood cell.

Please cite this article in press as: Hooda B et al. Effect of tranexamic acid on intraoperative blood loss and transfusion requirements in patients undergoing excision of intracranial meningioma. J Clin Neurosci (2017), http://dx.doi.org/10.1016/j.jocn.2017.02.053

Table 5
Extent of tumor removal, Quality of hemostasis and Post-operative hematoma.*

Parameter	Group T (n = 30)	Group P (n = 30)	p value
Extent of tumor removal			
Complete	24 (80)	26 (86.7)	0.49
Partial	6 (20)	4 (13.3)	
Grade of ooze			
0	2 (6.7)	1 (3.3)	0.007**
1	21 (70)	9 (30)	
2	5 (16.7)	14 (46.7)	
3	1 (3.3)	6 (20)	
4	1 (3.3)	0	
Hematoma (EDH/ SDH/Operative cavity bleed)	3 (10)	4 (13)	0.46

* Data given as number (%) of patients. EDH: extradural hematoma; SDH: subdural hematoma.
** Grade of hemostasis/Ooze: [Good grades 0–1 vs Poor grades ≥2].

Table 6
Postoperative Complications and Outcome in the two groups.*

Parameter	Group T (n = 30)	Group P (n = 30)	p value
Re-exploration	1 (3.3)	3 (10)	0.46
Hydrocephalus	0	1 (3.3)	
Seizures	1 (3.3)	2 (6.7)	
New neurologic deficit	4 (13.8)	10 (33.3)	0.12
Post-operative PRBC transfusion (ml)‡	500 (250,500) n = 3	750 (500,1000) n = 5	0.08
Duration of post-operative ventilation (h)‡	16 (11,38) n = 13	17 (12,226) n = 15	0.33
ICU stay (days)‡	1.5 (1, 7)	2 (1,10)	0.12
Hospital stay (days)‡	13 (4, 32)	15 (7, 31)	0.92
GOSE			0.49
Good recovery	25 (83.3)	21 (70)	
Moderate disability	4 (13.3)	7 (23.3)	
Severe disability/death	1 (3.4)	2 (6.7)	

* Data given as number (%) unless specified.
‡ Data given as median (Min, Max). PRBC: packed red blood cell; GOSE: glasgow outcome scale extended; ICU: intensive care unit.

dose of tranexamic acid used was arbitrary based on studies in spine surgery and CRASH-2 trial. Therefore, multiple dose comparative studies in large patient population are required to demonstrate its actual potential in reducing blood loss during intracranial tumor surgery.

5. Conclusion

Administration of tranexamic acid significantly reduced blood loss (by 27%) in patients undergoing elective craniotomy for excision of intracranial meningioma. Fewer patients required blood transfusion, and perioperative hemostasis was significantly better in tranexamic acid group. However, there was no significant difference in the amount of blood and blood products transfused, length of hospital stay and neurologic outcome. Therefore, large controlled trials with multiple dose comparisons are required to confirm the blood sparing effect of tranexamic acid in neurosurgical patients undergoing craniotomy for vascular tumors such as meningiomas and address safety concerns, if any, with its use.

Conflict of interest

Nil.

Appendix I: Assessment of oozing in surgical field (Modified from Boezaart et al.) [17].

Grades	Clinical features
0	Adequate hemostasis, virtually bloodless field
1	Minimal oozing, blood suctioning is not required
2	Moderate oozing requiring routine measures (occasional suctioning, cauterization, gel foam application)
3	Severe oozing requiring special efforts in addition to routine measures (fibrin glue, FloSeal, blood products)
4	Unsatisfactory surgical field despite all efforts requiring drainage system.

References

[1] Saraf S, Mccarthy BJ, Villano JL. Update on meningiomas. Oncologist 2011;16.1604-3.
[2] Hill JE, Frawley WH, Griffith KE, et al. Allogenic blood transfusion increases the risk of post operative bacterial infection: a meta-analysis. J Trauma 2003;54:908–14.
[3] Glance LG, Dick AW, Mukamel DB, et al. Association between intra-operative blood transfusion and morbidity in patients undergoing noncardiac surgery. Anesthesiology 2011;114:283–92.
[4] Goodnough LT, Shander A. Patient blood management. Anesthesiology 2012;116:367–76.
[5] Dunn CJ, Goa KL. Tranexamic acid: a review of its use in surgery and other indications. Drugs 1999;57:1005–32.
[6] McCormack PL. Tranexamic acid: a review of its use in hyperfibrinolysis. Drugs 2012;72:585–617.
[7] Adler SC, Brindle W, Burton G, et al. Tranexamic acid is associated with less blood transfusion in off-pump coronary artery bypass graft surgery: a systematic review and meta-analysis. J Cardiothorac Vasc Anesth 2011;25:26–35.
[8] Molenaar IQ, Warnaar N, Groen H, et al. Efficacy and safety of antifibrinolytic drugs in liver transplantation: a systematic review and meta-analysis. Am J Transplant 2007;7:185–94.
[9] Huang F, Wu D, Guangwen M, et al. The use of tranexamic acid to reduce blood loss and transfusion in major orthopedic surgery: a meta-analysis. J Surg Res 2014;186:318–27.
[10] Ker K, Edwards P, Perel P. Effect of tranexamic acid on surgical bleeding: systematic review and cumulative meta-analysis. Br Med J 2012;344:e3054.
[11] Henry DA, Carless PA, Moxey AJ, et al. Anti-fibrinolytic use for minimizing peri-operative allogeneic blood transfusion. Cochrane Database Syst Rev 2007;4:CD001886.
[12] Henry DA, Carless PA, Moxey AJ, et al. Anti-fibrinolytic use for minimizing peri-operative allogeneic blood transfusion. Cochrane Database Syst Rev 2011;3:CD001886.
[13] Wong J, El Beheiry H, Rampersaud YR, et al. Tranexamic acid reduces perioperative blood loss in adult patients having spinal fusion surgery. Anesth Analg 2008;107:1479–86.
[14] Roos YB, Germans MR, Rinkel GJ, et al. Antifibrinolytic therapy for aneurysmal subarachnoid haemorrhage. Cochrane Database Syst Rev 2000(2):CD001245.
[15] Hillman J, Fridriksson S, Nilsson O, et al. Immediate administration of tranexamic acid and reduced incidence of early rebleeding after aneurysmal subarachnoid hemorrhage: a prospective randomized study. J Neurosurg 2002;97:771–8.
[16] Vel R, Udupi BP, Satya Prakash MV, et al. Effect of low dose tranexamic acid on intra-operative blood loss in neurosurgical patients. Saudi J Anaesth 2015;9(1):42–8.
[17] Bolliger D, Seeberger MD, Tanaka KA. Principles and practice of thromboelastography in clinical coagulation management and transfusion practice. Transfus Med Rev 2012;26:1–13.
[18] Boezaart AP, van der Merwe J, Coetzee A. Comparison of sodium nitroprusside and esmolol induced controlled hypotension for functional endoscopic sinus surgery. Can J Anaesth 1995;42:373–6.
[19] American Society of Anesthesiologists task force on peri-operative blood transfusion and adjuvant therapies. Practice guidelines for peri-operative blood transfusion and adjuvant therapies. Anesthesiology 2006;105:198–208.
[20] Simpson D. The recurrence of intracranial meningiomas after surgical treatment. J Neurol Neurosurg Psychiatry 1957;20:22–39.
[21] Wilson JT, Pettigrew LE, Teasdale GM. Structured interviews for the glasgow outcome scale and the extended glasgow outcome scale: guidelines for their use. J Neurotrauma 1998;15:573–85.

Please cite this article in press as: Hooda B et al. Effect of tranexamic acid on intraoperative blood loss and transfusion requirements in patients undergoing excision of intracranial meningioma. J Clin Neurosci (2017), http://dx.doi.org/10.1016/j.jocn.2017.02.053

ARTICLE IN PRESS

[22] Ojemann RG. Surgical management of olfactory groove, suprasellar and medial sphenoid wing meningioma. In: Schmidek HH, editor. Meningiomas and their surgical management. Philadelphia: W. B. Saunders Co.; 1991. p. 242–59.
[23] Oka K, Tsuda H, Kamikaseda K, et al. Meningiomas and hemorrhagic diathesis. J Neurosurg 1988;69:356–60.
[24] Goh KY, Poon WS, Chan DT, et al. Tissue plasminogen activator expression in meningiomas and glioblastomas. Clin Neurol Neurosurg 2005;107:296–300.
[25] Gerlach R, Krause M, Seifert V, et al. Hemostatic and hemorrhagic problems in neurosurgical patients. Acta Neurochir 2009;151:873–900.
[26] Roberts I, Shakur H, Afolabi A, et al. The importance of early treatment with tranexamic acid in bleeding trauma patients: an exploratory analysis of the CRASH-2 randomised controlled trial. Lancet 2011;377:1096–101.
[27] The CRASH-2 Collaborators. Effect of tranexamic acid in traumatic brain injury: a nested randomised, placebo controlled trial (CRASH-2 Intracranial Bleeding Study). Br Med J 2011;343:d3795.
[28] Sander M, Spies CD, Martiny V, et al. Mortality associated with administration of high-dose tranexamic acid and aprotinin in primary open-heart procedures: a retrospective analysis. Crit Care 2010;14:R148.
[29] Fergusson DA, Hébert PC, Mazer CD, et al. A comparison of aprotinin and lysine analogues in high risk cardiac surgery. N Eng J Med 2008;358:2319–31.
[30] Fodstad H, Forssell A, Liliequist B, et al. Antifibrinolysis with tranexamic acid in aneurysmal subarachnoid hemorrhage: a consecutive controlled clinical trial. Neurosurgery 1981;8:158–65.
[31] Ortmann E, Besser MW, Klein AA. Antifibrinolytic agents in current anaesthetic practice. Br J Anaesth 2013;110:1–15.
[32] Murkin JM, Falter F, Granton J, et al. High-dose tranexamic acid is associated with non ischemic clinical seizures in cardiac surgical patients. Anesth Analg 2010;110:350–3.

Please cite this article in press as: Hooda B et al. Effect of tranexamic acid on intraoperative blood loss and transfusion requirements in patients undergoing excision of intracranial meningioma. J Clin Neurosci (2017), http://dx.doi.org/10.1016/j.jocn.2017.02.053
