# Supplementary material for: Efficacy and safety of tranexamic acid on blood loss and seizures in patients undergoing meningioma resection: A systematic review and meta-analysis
Source: PLoS One. 2024 Sep 4;19(9):e0308070. doi: 10.1371/journal.pone.0308070 (PMC11373793; doi:10.1371/journal.pone.0308070)
Supplement: S1 File — (ZIP) [file pone.0308070.s004.zip › 2023-JCA-TXA-Cofirst, TXA.pdf]

Journal of Clinical Anesthesia 92 (2024) 111285

Contents lists available at ScienceDirect

Journal of Clinical Anesthesia

journal homepage: www.elsevier.com/locate/jclinane

Original Contribution

Intravenous tranexamic acid for intracerebral meningioma resections: A randomized, parallel-group, non-inferiority trial

Shu Li, M.D., Ph.D [a,1], Minying Liu, M.D [a,1], Jingchao Yang, M.D [b], Xiang Yan, M.D [c], Yaru Wu, M.D [a], Liyong Zhang, M.D [a], Min Zeng, M.D., Ph.D [a], Dabiao Zhou, M.D., Ph.D [d], Yuming Peng, M.D., Ph.D [a,e,*], Daniel I. Sessler, M.D [f,e]

[a] *Department of Anesthesiology, Beijing Tiantan Hospital, Capital Medical University, Beijing, PR China*
[b] *Department of Anesthesiology, Cancer Hospital, Chinses Academy of Medical Sciences, Beijing, PR China*
[c] *Department of Anesthesiology, Beijing Chao-Yang Hospital, Capital Medical University, Beijing, PR China*
[d] *Department of Neurosurgery, Beijing Tiantan Hospital, Capital Medical University, Beijing, PR China*
[e] *Outcome Research Consortium, Cleveland, OH, USA*
[f] *Department of Outcome Research, Cleveland Clinic, Cleveland, OH, USA*

HIGHLIGHTS

- Tranexamic acid could reduce surgical bleeding but occasionally causes seizures.
- A single dose of 20 mg/kg of tranexamic acid is non-inferior regarding postoperative seizure with a risk difference of 0.7%.
- NThe single dose of tranexamic acid also did not significantly reduce bleeding.
- Other complications, blood loss, hemoglobin level changes, and blood product transfusion were also similar.

ARTICLE INFO

Keywords:
Anesthesia
Brain tumor resection
Adult
Antifibrinolytic
Postoperative seizure
Tranexamic acid
Non-inferiority

ABSTRACT

Study objectives: Tranexamic acid (TXA) is an antifibrinolytic that is widely used to reduce surgical bleeding. However, TXA occasionally causes seizures and the risk might be especially great after neurosurgery. We therefore tested the hypothesis that TXA does not meaningfully increase the risk of postoperative seizures within 7 days after intracranial tumor resections.
Design: Randomized, double-blind, placebo-controlled, non-inferiority trial.
Setting: Beijing Tiantan Hospital, Capital Medical University.
Patients: 600 patients undergoing supratentorial meningioma resection were included from October 2020 to August 2022.
Interventions: Patients were randomly assigned to a single dose of 20 mg/kg of TXA after induction ($n = 300$) or to the same volume of normal saline (n = 300).
Measurement: The primary outcome was postoperative seizures occurring within 7 days after surgery, analyzed in both the intention-to-treat and per-protocol populations. Non-inferiority was defined by an upper limit of the 95% confidence interval for the absolute difference being <5.5%. Secondary outcomes included incidence of non-epileptic complication within 7 days, changes in hemoglobin concentration, estimated intraoperative blood loss. Post hoc analyses included the types and timing of seizures, oozing assessment, and a sensitivity analysis for the primary outcome in patients with pathologic diagnosis of meningioma.
Main results: All 600 enrolled patients adhered to the protocol and completed the follow-up for the primary outcome. Postoperative seizures occurred in 11 of 300 (3.7%) of patients randomized to normal saline and 13 of 300 (4.3%) patients assigned to tranexamic acid (mean risk difference, 0.7%; 1-sided 97.5% CI, $-\infty$ to 4.3%; $P =$

* Corresponding author at: Department of Anesthesiology, Beijing Tiantan Hospital, Capital Medical University, No.119, Nansihuan Xilu, Fengtai District, Beijing 100070, PR China.
E-mail addresses: lishu@bjtth.org (S. Li), zengmin@bjtth.org (M. Zeng), zhoudabiao@bjtth.org (D. Zhou), pengyuming@bjtth.org (Y. Peng), DS@CCF.org (D.I. Sessler).
[1] Shu Li and Minying Liu contributed equally.

https://doi.org/10.1016/j.jclinane.2023.111285
Received 22 May 2023; Received in revised form 14 September 2023; Accepted 8 October 2023
0952-8180/© 2023 Published by Elsevier Inc.

0.001 for noninferiority). No significant differences were observed in any secondary outcome. Post hoc analysis indicated similar amounts of oozing, calculated blood loss, recurrent seizures, and timing of seizures.

Conclusion: Among patients having supratentorial meningioma resection, a single intraoperative dose of TXA did not significantly reduce bleeding and was non-inferior with respect to postoperative seizures after surgery.

Registry information: This trial was registered at clinicaltrials.gov (NCT04595786) on October 22, 2020, by Dr. Yuming Peng.

1. Introduction

Tranexamic acid (TXA) is an antifibrinolytic drug that promotes hemostasis across of range of settings. [1–4] For example, the drug reduces bleeding in a wide variety of noncardiac and cardiac procedures both at low and high doses. [3,5–7] The drug is minimally toxic, but can promote seizures. In patients with cerebral hemorrhage, seizures were the most common safety outcome but the incidence was similar at 7% in patients assigned to TXA and placebo. [6] In contrast, seizures were slightly more common when patients having non-cardiac surgery were assigned to TXA. [7] Moreover, the risk of seizure appears to be dose-dependent in patients having cardiac surgeries. [5]

Postoperative seizures are common after intracranial procedures and often progress to chronic postoperative epilepsy with potentially severe consequences [8–10] including persistent neurological morbidity, impaired life quality, and prolonged hospitalization. Observational studies also indicate that postoperative seizures are strongly associated with epilepsy requiring chronic pharmacologic treatment. [9,11] Postoperative seizures are, therefore, the major safety concern related to the use of TXA in patients having intracranial surgery.

TXA is thought to provoke seizures by inhibiting γ-aminobutyric acid type A receptors and glycine receptors, both of which increase the excitability of neuronal networks. [12,13] Patients having surgery for brain tumors are at special risk because disruption of the blood-brain barrier facilitates entry of TXA into the central nervous system. [14] Furthermore, neurosurgical patients inevitably experience cortical trauma and edema, disruption of the blood-brain barrier, and changes in microcirculation during craniotomy [15,16] — all of which promote postoperative seizures. Neurosurgical patients are thus already at high risk of seizures, and TXA likely further increases risk.

Both bleeding control and seizure prevention are both important in neurosurgery. [17,18] TXA helps maintain hemostasis in traumatic brain injury and spinal surgery, [19–21] but whether the drug promotes postoperative seizures in neurosurgical patients remains unclear because available studies are too small to evaluate this relatively rare complication. [22–24] We, therefore, tested the hypothesis that a single dose of TXA(20 mg/kg) is non-inferior to placebo with respect to seizures within 7 days after supratentorial meningioma resections.

2. Materials and methods

This randomized, parallel-group, placebo-control, non-inferiority trial was conducted at Beijing Tiantan Hospital, Capital Medical University. Patients were enrolled from October 2020 to August 2022. The trial protocol was approved by the Chinese Ethics Committee of Registering Clinical Trials (ChiECRCT20200224).

The trial was registered at ClinicalTrials.gov (NCT04595786, Principal Investigator Yuming Peng) on October 22, 2020. Written consent was obtained from each participating patient or his/her legal representatives. This report follows the Consolidated Standards of Reporting Trials recommendations for presenting clinical trials. The full study protocol was previously published. [25]

2.1. Subject selection

We considered consenting patients aged 18–80 years who had a radiological diagnosis of supratentorial meningioma, were scheduled for elective craniotomy under general anesthesia and were designated American Society of Anesthesiologists physical status of I to III. We excluded patients who were allergic to TXA, who had a history of seizures or thrombotic disease, chronic kidney disease (glomerular filtration rate < 60 mL/min or albumin–creatinine ratio > 30 mg/g), [26] or were breastfeeding or pregnant.

2.2. Randomization and masking

Patients were randomized before surgery in a 1:1 ratio by an independent research assistant based on a computer-generated randomized table with block sizes of 4 or 6. Allocation was concealed by having masked trial drug (20 mg/kg TXA or comparable volume of normal saline) delivered to clinicians by a research nurse in identical-appearing 50-mL syringes. Thereafter, all anesthesiologists, neurosurgeons, and endpoint assessors were blinded to treatment. The TXA we used was manufactured by Shanxi Pude Pharmaceutical Co, Ltd., Shanxi, China.

2.3. Trial intervention

The trial drug was infused over 20 min to a total of 20 mg/kg TXA (or comparable volume of saline). The dose of TXA was selected to be both effective and safe based on previous observational studies and small randomized trials. [6,24,27–30] For example, a single dose of 20 mg/kg TXA provides a plasma concentration ≥ 10 mg/L for 4 h which substantially impairs fibrinolysis. [31–34] Our other consideration was a meta-analysis reporting a dose-dependent increase in the risk of seizures in patients given >2 g/day of TXA (risk ratio 3.05; 95% CI: 1.01–9.20). [35]

2.4. Concomitant treatments

Patients were premedicated with midazolam (0.05 mg/kg) intravenously 5 min before anesthetic induction. Sufentanil (0.3–0.4 μg/kg) and propofol (1.5–2.5 mg/kg) were given for anesthetic induction, followed by rocuronium (0.9 mg/kg) or cisatracurium (0.2 mg/kg) for muscle relaxation. After tracheal intubation, mechanical ventilation was adjusted to maintain normocapnia with a tidal volume of 6–8 mL/kg, a respiratory rate of 12–15 breaths per min, an inspiration/expiration (I:E) ratio of 1:2 and 50% inspired oxygen at a fresh-gas flow of 1–2 L/min. Anesthesia was maintained with combined intravenous and inhalation anesthesia, with propofol (3–8 mg/kg/h), remifentanil (0.1–0.2 μg/kg/min), and volatile anesthesia not exceeding 0.5 minimal alveolar concentration, to target bispectral (BIS) index values between 40 and 50. [36] Intraoperative mean arterial pressures were targeted to ±20% of the baseline value.

Allogeneic erythrocytes, fresh frozen plasma, platelets, cryoprecipitate, and prothrombin complex concentrate were infused according to the blood transfusion guideline. [37] Cell salvage was used when the estimated blood loss exceeded 500 mL. [38] Intraoperative cranial nerve blocks were performed according to incision location and anesthesiologists' preference. An intraoperative prophylactic antiepileptic was initiated after tumor resection if requested by the surgeon. [39–42]

Patients recovered in the Post Anesthesia Care Unit (PACU), an intensive care unit (ICU), or a neurosurgical ward. Patients were not normally given antiepileptic drugs in the PACU unless a seizure was

observed. Other administered drugs were recorded, including seizure prophylaxis.

2.5. Trial end points

Our objective was to evaluate the effect of a single dose of TXA on seizures during the initial 7 postoperative days. Seizures were defined by transient involuntary movements, abnormal sensory phenomena, or an otherwise unexplained altered mental status observed by the neurosurgeons, intensive care staff, care workers, and documented in the medical records. [10] Seizure classifications and subtypes were assigned per definitions of the International League Against Epilepsy (ILAE) and were recorded. [43] When seizure-like activity was of unclear origin, electroencephalograms (EEG) and brain CTs were conducted within 24 h to confirm or refute the diagnosis, and to identify other possible causes of mental status alteration. We considered events to be seizures only if the clinical presentation was clear or there was EEG evidence of interictal or seizure activity. All assessors were fully blinded to treatment.

Our secondary outcomes were cumulative complications within 7 days, changes in hemoglobin concentrations, estimated intraoperative blood loss, intraoperative blood product transfusions, and rate, and cell salvage volume and rate. On a post hoc exploratory basis, we considered the effect of TXA on postoperative seizure subtypes, recurrent seizures, and time to onset of postoperative seizures within 7 days, as well as on surgical field oozing.

Postoperative complications included hydrocephalus, cerebral edema, intracranial hematoma, deep vein thrombosis, pulmonary embolism, cerebral venous sinus thrombosis, cerebral ischemia stroke, myocardial infarction, acute kidney infarction, anemia (hemoglobin $<$13 g/dL for men and $<$ 12 g/dL for women), infection, and reoperation (Supplementary Table 1). Changes in hemoglobin concentrations were calculated as the difference between baseline hemoglobin concentration and the first hemoglobin concentration recorded postoperatively day 1. Estimated intraoperative blood loss was calculated as collected blood volume in the suction canister (mL) − the volume of flush (mL) + volume from gauze sponges (mL) [44]. We assumed that cell salvage efficiency was 50%, and the estimated blood loss was calculated using the formula: [(250 mL/bowl) * number of bowls] / 50% + volume from gauze sponges. [45] We also estimated blood loss using the method described by López-Picado A. [46,47]

Oozing in the surgical field was assessed using a 0–4-point surgeon-graded subjective scale, with 4 points representing an unsatisfactory surgical field despite all efforts, and requiring a drainage system. [24] We also recorded hospital length of stay, length of ICU stay, unplanned ICU admission, reoperation, postoperative mechanical ventilation, hospitalization cost, and all-cause mortality at 7 days.

2.6. Sample size estimation

In an observational study in our institution, the incidence of postoperative seizure after supratentorial meningioma resections was 3.8%. [8] Other observational studies report incidences of postoperative seizures of 4.8%, 6.4%, and 8.6%. [9,10,36] We assumed that the incidence of new-onset seizures within 7 days after meningioma resection would be 6.0% in the placebo group, which was the average frequency in the reference group of these published studies.

We selected an absolute noninferiority margin of 5.5% based on a meta-analysis indicating that low-dose TXA (<45 mg/kg) introduces a 4.7–5.4-fold increase in the odds of postoperative seizures. [48] Consequently, the TXA group would be declared non-inferior if the upper limit of 95% confidence interval (CI) for the difference was below the noninferiority margin. With α set at a one-sided 0.025 and a dropout rate of 2.5%, a sample size of 600 patients (300 per group) would provide 80% power.

2.7. Statistical analysis

The trial protocol including the statistical analysis plan was published [25] and submitted to the Ethics Boards before enrolling patients. Consequently, the statistical analysis plan was developed before data were accessed. Post hoc analyses are specified.

Baseline characteristics are presented as numbers and percentages in TXA and normal saline patients, along with absolute standardized differences which are defined as absolute differences in means, mean ranks, or proportions divided by the pooled standard deviation. Variables with absolute standardized difference > 0.160 ($=1.96^*\sqrt{\frac{1}{300}+\frac{1}{300}}$, where 300 was the number of patients in each group), were considered imbalanced and adjusted for in the primary outcome analysis.

Continuous variables were compared with Student *t*-tests when normally distributed or otherwise with Mann-Whitney tests, and the categorical variables were compared with χ^2 tests. For the primary outcome, postoperative seizure within 7 days, we analyzed completed cases with 7-day follow-up data in the per-protocol set.

A χ^2 test was used to compare the primary endpoints across the 2 groups. Missing data were imputed using inverse probability weighting and the worst-case imputation scenarios. Secondary outcomes were analyzed by Student t, Mann-Whitney, or chi-square tests as appropriate. As usual for non-inferiority comparisons, the nominal significance level was a 1-tailed $P < 0.025$ for the primary outcome. All other comparisons were based on 2-sided $P < 0.05$. We did not compensate for multiple secondary and post hoc comparisons so these analyses should be considered exploratory. All tests were performed with STATA V.15.0 (StataCorp).

Pre-specified subgroup analyses were performed on age, sex, peritumor edema, tumor site, and tumor volume using interaction terms in regression models. $P < 0.05$ was considered statistically significant for treatment-by-covariate subgroup interactions.

On a post hoc basis, we explored the time to seizure onset with Kaplan-Meier cumulative incidence curve and a log-rank test to compare hazards in each treatment group. Subtype and classification of early postoperative seizure were also compared between groups. Sensitivity analyses were also performed in patients with postoperative pathologically confirmed meningioma patients.

3. Results

From October 2020 to August 2022, 955 patients who had meningioma resections at Beijing Tiantan Hospital, Capital Medical University were assessed for eligibility. Among them, 355 were excluded and 600 (mean age 53 years, 72% female) were enrolled and randomized to the TXA group or normal saline group.

Overall, demographic and baseline variables were well-balanced between the two groups, and there were no notable differences in baseline characteristics (Table 1) or surgical characteristics (Table 2). Two hundred and ninety patients in the TXA group and 287 patients in the normal saline group were pathologically confirmed as meningioma postoperatively. The remaining patients were with hemangiopericytoma, cavernous malformation, neurofibromatosis, schwannoma, hypophysoma, epithelioid glioblastoma, Rosai-Dorfman disease, and olfactory neuroblastoma. On average, patients in the TXA group were given 1.3 g (IQR 1.2 to 1.5 g) of TXA; only two patients were given >2 g.

3.1. Primary outcome

One hundred and forty patients were discharged before day 7 at a median postoperative duration of 6 days (IQR 5 to 6 days). The primary outcome in those patients was assessed by phone. The primary outcome was therefore available for all patients and no missing data were imputed (Fig. 1). Postoperative seizures during the initial postoperative 7 days were observed in 13 (4.3%) patients randomized to the TXA

Table 1
Demographic and clinical characteristics at baseline.

	Normal saline (n = 300)	Tranexamic acid (n = 300)	Absolute standardized differences
Age, median (interquartile range year	53 (46, 61)	53 (44, 61)	0.054
Sex, female, No. (%)	225 (75.0)	205 (68.3)	0.148
Body mass index, median (interquartile range) [a]	24.4 (22.5, 26.8)	24.2 (22.1, 26.8)	0.036
Medical history, No. (%)			
History of smoking	53 (17.7)	61 (20.3)	0.068
History of drinking	75 (25.0)	75 (25.0)	0.000
Hypertension	69 (23.0)	83 (27.7)	0.107
Diabetes	24 (8.0)	34 (11.3)	0.113
Cardiac disease	7 (2.3)	11 (3.7)	0.078
Stroke or TIA	4 (1.3)	5 (1.7)	0.024
Pulmonary disease	6 (2.0)	3 (1.0)	0.082
Kidney dysfunction	3 (1.0)	1 (0.3)	0.082
Liver dysfunction	7 (2.3)	6 (2.0)	0.023
Allergic history	40 (13.3)	49 (16.3)	0.084
Chemotherapy/radiotherapy	6 (2.0)	3 (1.0)	0.082
TBI history	22 (7.3)	15 (5.0)	0.097
Pre-operative medication, No. (%)			
Mannitol	5 (1.7)	11 (3.7)	0.124
Antiepileptic	6 (2.0)	14 (4.7)	0.149
Estrogen	2 (0.7)	0 (0.0)	0.116
Neurological symptom, No. (%)			
Headache	89 (29.7)	111 (37.0)	0.156
Vomiting or Nausea	11 (3.7)	16 (5.3)	0.080
Motor/sensory dysfunction	23 (7.7)	25 (8.3)	0.031
Aphasia	6 (2.0)	6 (2.0)	0.000
Cranial nerve deficit	53 (17.7)	54 (18.0)	0.009
Tumor characteristics			
Tumor site, No. (%)			
Convexity	150 (50.0)	159 (53.0)	0.060
Others	150 (50.0)	141 (47.0)	
Side of lesions, No. (%)			
Left	128 (43.1)	134 (45.4)	0.036
Right	140 (47.1)	133 (45.1)	
Crossing midline	29 (9.8)	28 (9.5)	
Major cerebral artery involvement, No. (%)	69 (23.0)	58 (19.3)	0.015
Sinus or major venous involvement, No. (%)	90 (30.0)	88 (29.3)	0.090
Tumor equivalent diameter, median (interquartile range), mm	30.7 (22.0, 41.3)	30.0 (23.0, 41.6)	0.024
Midline shift, median (interquartile range), mm	0 (0, 5)	0 (0, 4)	0.120
Tempo-frontal lobe involvement, No. (%)	104 (34.7)	88 (29.3)	0.114
Recurrent tumor, No. (%)	18 (6.0)	11 (3.7)	0.109
Tumor type, No. (%)			
Meningioma	287 (95.7)	290 (96.7)	0.052
Others [b]	13 (4.3)	10 (3.3)	
World Health Organization classification, No. (%)			
I-II	290 (96.7)	293 (97.7)	0.107
III-IV	6 (2.0)	7 (2.3)	
Unknown	4 (1.3)	0 (0.0)	
Preoperative assessment			
ASA classification, No. (%)			
I	10 (3.3)	11 (3.7)	
II	194 (64.7)	189 (63.0)	0.019
III	96 (32.0)	100 (33.3)	
Glasgow Coma scale, median (interquartile range)	15 (15, 15)	15 (15, 15)	0.024
Charlson Comorbidity Index, median (interquartile range)	1 (0,2)	1 (0, 2)	0.026
Karnofsky performance score, median (interquartile range)	90 (80, 90)	90 (80, 90)	0.008
Caprini score, median (interquartile range)	1 (1,2)	1 (1, 2)	0.018
Venous thromboembolism risk, No. (%)[c]			
Very low risk (Caprini risk score = 0)	28 (9.3)	33 (11.0)	0.076
Low (Caprini risk score 1–2)	235 (78.3)	235 (78.3)	
Moderate (Caprini risk score 3–4)	35 (11.7)	31 (10.3)	
High (Caprini risk score > 5)	2 (0.7)	1 (0.3)	
Pre-operative baseline laboratory values			
APTT, median (interquartile range), s	30.6 (28.6, 33.1)	31.0 (29.3, 32.4)	0.051
Fbg, median (interquartile range), g L^{-1}	2.86 (2.57, 3.24)	2.80 (2.48, 3.18)	0.113
Hemoglobin concentration, median (interquartile range), g dL^{-1}	14.0 (13.1, 14.9)	14.1 (13.1, 15.2)	0.063
Platelet count, median (interquartile range), 10ˆ9 L^{-1}	240 (206, 282)	236 (195, 276)	0.110

TIA, transient ischemic attack; TBI, traumatic brain injury; ASA, American Society of Anesthesiologists.

[a] Calculated as weight in kilograms divided by height in meters squared.

[b] Others include hemangiopericytoma, cavernous malformation, neurofibromatosis, schwannoma, hypophysoma, epithelioid glioblastoma, Rosai-Dorfman disease, and olfactory neuroblastoma.

[c] The Caprini risk score of 0 is defined as very low risk, a 1–2 score is defined as low risk, a 3–4 score is defined as moderate risk and a higher or equal to 5 score is defined as high risk.

group and 11 (3.7%) patients allocated to normal saline placebo. The TXA group was non-inferior to the normal saline group for postoperative seizures (risk difference, 0.7%; 1-sided 97.5% CI, $-\infty$ to 4.3%; $P = 0.001$ for noninferiority, Table 3).

TXA did not consistently increase the incidence of postoperative seizure across various pre-defined subgroups, nor were significant interactions identified (Fig. 2). About half the seizures in each group were focal and half generalized (Table 3). Most seizure patients had only a single episode; however, 4 (1.3%) patients given TXA and 3 (1.0%) patients given normal saline group had multiple episodes.

In the sensitivity analysis of pathological diagnosis as meningioma, 13 patients (4.5%) in the TXA group and 11 patients (3.8%) in the normal saline group experienced a seizure (risk difference 0.7%; 1-sided 97.5% CI, $-\infty$ to 3.9%; $P = 0.002$ for noninferiority), respectively.

3.2. Secondary outcomes

Overall, 26 (8.7%) in the TXA group and 22 patients (7%) in the normal saline group experienced thrombotic events (risk difference 1.3%; 95% CI, −3.0 to 5.7, $P = 0.547$). One patient assigned to the normal saline group experienced both pulmonary embolism and deep

Table 2
Intraoperative and postoperative characteristics.

	Normal saline (n = 300)	Tranexamic acid (n = 300)	*P* value
Intraoperative variables			
Surgery duration, median (interquartile range), hour	3.5 (2.7, 4.8)	3.5 (2.6, 4.5)	0.246
Anesthesia duration, median (interquartile range), hour	4.5 (3.7, 5.9)	4.5 (3.5, 5.4)	0.161
Multimodal analgesia, No. (%) [a]	267 (89.0)	272 (90.7)	0.499
Central venous catheter, No. (%)	97 (32.3)	89 (29.7)	0.480
Intraoperative medications, No. (%)			
Antiepileptic	114 (38.0)	126(42.0)	0.317
Mannitol	174 (58.0)	165 (55.0)	0.459
Glucocorticoid	77 (25.7)	80 (26.7)	0.781
Vasopressor	102 (34.0)	101 (33.7)	0.931
Simpson grade of meningioma resection, No. (%)			0.496
0	26 (9.1)	27 (9.3)	
1	161 (56.1)	167 (57.6)	
2	79 (27.5)	76 (26.2)	
3	4 (1.4)	9 (3.1)	
4	17 (5.9)	11 (3.8)	
5	0 (0.0)	0 (0.0)	
Adjuvant techniques and technologies, No. (%) [b]	9 (3.0)	9 (3.0)	1.000
Intraoperative fluid and hemodynamic			
Total infusion, median (interquartile range), mL	2500 (2000,3300)	2500 (2000,3100)	0.994
Urine, median (interquartile range), mL	1180 (800, 1700)	1200 (800, 1600)	0.731
Postoperative treatment, No. (%)			
Antiepileptic [c]	295 (98.3)	297 (99.0)	0.408
Dehydration treatment [d]	290 (96.7)	283 (94.3)	0.168
Discharge before day 7, No. (%)	67 (22.3)	73 (24.3)	0.562

[a] Multimodal analgesia is composed of scalp nerve block, incision infiltration, and intravenous patient-controlled analgesia.
[b] Adjuvant techniques and technologies include intraoperative ultrasound imaging, intraoperative neurophysiologic monitoring, neuro-navigation, and indocyanine green video angiography to identify tumor remnants, functional cortex, and feeding arteries.
[c] Postoperative antiepileptic treatment includes intravenous or oral administration of valproic acid, oxcarbazepine, carbamazepine, levetiracetam, and phenobarbital. Rescue intramuscular phenobarbital or continuous infusion of midazolam was administered when seizure fit attacks.
[d] Postoperative dehydration treatment includes intravenous administration of 20% mannitol, dexamethasone, and methylprednisolone.

vein thrombosis. Four patients (1.3%) in the TXA and 1 patient (0.3%) in the normal saline group experienced ischemic events (risk difference 1.0%; 95% CI, −0.5 to 2.5, $P = 0.178$), and similarly, one patient in the TXA with myocardial infarction and cerebral infarction at the same time. Other non-epileptic complications, including hematoma, hydrocephalus, cerebral edema, infection, and reoperation within 7 days after surgery, were generally similar between groups (Table 3).

One hundred and eighty-two (61%) patients in the normal saline group and 161 (54%) patients in the TXA group experienced postoperative anemia (hemoglobin <13 g/dL for men and < 12 g/dL for women) (risk difference, −7.0%; 95% CI, −14.9% to 0.9%]), a difference that was not statistically significant ($P = 0.083$). The median estimated intraoperative blood loss was 250 mL (IQR, 150 to 500 mL) and 250 mL (IQR, 200 to 500 mL) in the TXA group and the placebo group, with a mean difference of -8 mL (95% CI -88 to 73 mL, $P = 0.505$). Changes in hemoglobin concentrations were also comparable between groups (mean difference 0.1 g/dL, 95% CI -0.1 to 0.2 g/dL, $P = 0.547$). The median amount of allogeneic erythrocyte transfusion was 0 (IQR, 0 to 0) mL in both groups (mean difference, 7 mL; 95%CI, −14 to 28 mL; $P = 0.554$), while the mean difference of fresh frozen plasma volume was −4 (95%CI -23 to 15 mL; $P = 0.975$) mL. No patients were given intraoperative cryoprecipitate or prothrombin complex concentrate. Cell saver use and volume were comparable in each group (Table 3).

There was no statistically significant difference between groups in the median length of hospital stay, length of ICU stays, hospitalization cost, unplanned ICU admission, postoperative mechanical ventilation, or reoperation (Table 3). The 7-day Karnofsky Performance Scale score, Glasgow coma score, and mortality were comparable in each group (Table 3).

3.3. *Post hoc analyses*

The hazard ratio for postoperative seizures over the initial 7 postoperative days was 1.20 (95% CI, 0.53 to 2.67; $P = 0.663$, Table 3, Supplementary Fig. 1). Moreover, subtypes of postoperative seizure ($P = 0.884$) and recurrent seizures ($P = 0.704$) were similar in each group. Calculated blood loss and the amount of surgical oozing were also similar in each group (Table 3).

4. Discussion

The overall incidence of seizures within 7 days after supratentorial meningioma resections was 4% which was less than the incidence we anticipated based on previous publications in similar patients. However, the 3.7% incidence of seizures in our placebo patients was nearly identical to the 3.8% incidence we previously reported. [8] As might be

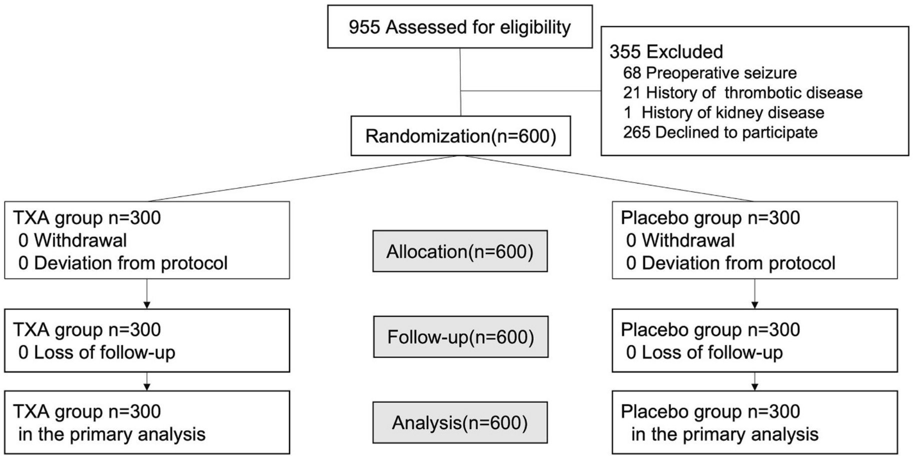

Fig. 1. Trial flow chart.

Table 3
Effectiveness outcomes.

	Normal saline (n = 300)	Tranexamic acid (n = 300)	Estimate of difference, hazard ratio ✢ (95% CI)	*P* values
Primary outcome				
Incidence of postoperative seizure within 7 days, No. (%)	11 (3.7)	13 (4.3)	0.7 (−∞ to 4.3) [a]	**0.001**
Secondary outcomes				
Non-epileptic complications within 7 days, No. (%)				
Anemia	182 (60.7)	161 (53.7)	−7.0 (−14.9 to 0.9)	0.083
Venous thromboembolism	22 (7.3)	26 (8.7)	1.3 (−3.0 to 5.7)	0.547
Deep vein thrombosis	22 (7.3)	26 (8.7)	1.3 (−3.0 to 5.7)	0.547
Pulmonary embolism	1 (0.3)	0 (0.0)	−0.3 (−1.0 to 0.3)	0.317
Ischemic events	1 (0.3)	4 (1.3)	1.0 (−0.5 to 2.5)	0.178
Myocardial infarction	0 (0.0)	2 (0.7)	0.7 (−0.3 to 1.6)	0.157
Cerebral infarction	1 (0.3)	3 (1.0)	0.7 (−0.6 to 2.0)	0.316
Hematoma	5 (1.7)	3 (3.0)	−0.7 (−2.5 to 1.2)	0.477
Hydrocephalus	2 (0.7)	0 (0.0)	−0.7 (−1.6 to 0.3)	0.157
Cerebral edema	1 (0.3)	0 (0.0)	−0.3 (−1.0 to 0.3)	0.317
Infection [*]	28 (9.3)	26 (8.7)	−0.7 (−5.2 to 3.9)	0.775
Estimated intraoperative blood loss, median (interquartile range), mL	250 (200, 500)	250(150, 500)	−8 (−88 to 73)	0.505
Changes in hemoglobin concentrations, median (interquartile range), g dL^{-1}	1.3 (0.6, 1.8)	1.3 (0.4, 1.9)	0.1 (−0.1 to 0.2)	0.547
Volume of transfusion, median (interquartile range)				
Allogeneic erythrocyte, mL	0 (0,0)	0 (0, 0)	7 (−14 to 28)	0.554
Fresh frozen plasma, mL	0 (0, 0)	0 (0, 0)	−4 (−23 to 15)	0.975
Platelets, mL	0 (0, 0)	0 (0, 0)	0 (0 to 0)	0.317
Patients with transfusion, No. (%)				
Allogeneic erythrocyte	17 (5.7)	14 (4.7)	−1.0 (−4.5 to 2.5)	0.580
Fresh frozen plasma	17 (5.7)	17 (5.7)	0.0 (−3.7 to 3.7)	1.000
Platelets	0 (0.0)	1 (0.3)	0.3 (−0.3 to 1.0)	0.317
Cell saver use, No. (%)	60 (20.0)	51 (17.0)	−3.0 (−9.2 to 3.2)	0.344
Volume of cell saver, median (interquartile range), mL	0.0 (0.0, 0.0)	0.0 (0.0, 0.0)	9.9 (−24.4 to 44.3)	0.336
Others				
Length of hospital stay, median (interquartile range), day	11.0 (8.5, 13.0)	10.0 (8.0, 13.0)	−0.2 (−0.9 to 0.6)	0.978
Length of ICU stay, median (interquartile range), day	0.0 (0.0,1.0)	0.0 (0.0, 1.0)	0.2 (−0.3 to 0.7)	0.394
Hospitalization cost, median (interquartile range), thousand CNY	54.0 (46.0, 64.0)	54.0 (47.0, 64.5)	1.1 (−3.7 to 5.9)	0.845
Reoperation, No. (%)	6 (2.0)	3 (1.0)	−1.0 (−2.9 to 0.9)	0.314
Unplanned intensive care unit admission, No. (%)	7 (2.3)	10 (3.3)	1.0 (−1.7 to 3.7)	0.460
Mechanical ventilation, No. (%)	5 (1.7)	3 (1.0)		
7-day Karnofsky performance score, median (interquartile range)	70.0 (70.0, 80.0)	70.0 (70.0, 80.0)	−0.5 (−2.8 to 1.8)	0.488
7-day Glasgow coma scale, median (interquartile range)	15.0 (15.0, 15.0)	15.0 (15.0, 15.0)	0.0 (−0.2 to 0.2)	0.655
7-day mortality after surgery, No. (%)	0 (0.0)	0 (0.0)	/	1.000
Exploratory analyses				
Assessment of oozing in surgical field, NO. (%)				
0–2	283 (94.3)	288 (96.0)		0.341
3–4	17 (5.7)	12 (4.0)		
Calculated blood loss, median (interquartile range)[b], mL	510 (311, 682)	514 (327, 687)	4 (−74 to 81)	0.992
Time to unset of postoperative seizure within, median (interquartile range), day	2 (1,3)	2 (1, 3)	1.20 (0.53 to 2.67) ✢	0.663
Type of postoperative seizure within 7 days, No. (%)				0.844
No seizure attack	289 (96.3)	287 (95.7)	/	
Focal onset	6 (2.0)	6 (2.0)	0.0 (−2.2 to 2.2)	1.000
Generalized onset	5 (1.7)	7 (2.3)	0.7 (−1.6 to 2.9)	0.560
Recurrent seizure, No. (%)	3 (1.0)	4 (1.3)	0.3 (−1.4 to 2.1)	0.704
Subcategories of postoperative seizure within 7 days, No. (%)				0.295
Tonic-Clonic	3/11 (27.3)	4/13 (30.8)	/	
Clonic	4/11 (36.4)	5/13 (38.5.9)	/	
Myoclonic	0/11 (0.0)	2/13 (15.4)	/	
Tonic	0/11 (0.0)	1/13 (7.7)	/	
Absence	4/11 (36.4)	1/13 (7.7)	/	

✢ Hazard ratio with 95% confidence interval Cox regression.
[*] Including post-craniotomy meningitis and respiratory infection.
[a] One-sided 97.5%CI; noninferiority test with 5%margin.
[b] Calculated blood loss is calculated using López-Picado's formula that blood loss = [estimated blood volume×(Hct_i − Hct_f) + transfused RBC volume]/Hct_{mean}, where estimated blood volume is determined using the ICSH formula, Hct_i is the initial hematocrit at the beginning of surgery, Hct_f is the final hematocrit at closure, and Hct_{mean} is the mean hematocrit (between initial and final).

Subgroups	Noraml saline	Tranexamic acid	Risk difference, % (95% CI)	Risk Ratio (95% CI)	P value for interation
	No. of event (%)				
**Age**					0.106
Age<=50	6 (4.9)	3 (2.4)	-2.50 (-7.10 to 2.20)	0.49 (0.13 to 1.92)	
Age>50	5 (2.8)	10 (5.7)	2.89 (-1.33 to 7.11)	2.02 (0.71 to 5.80)	
**Sex**					0.344
Male	3 (4.0)	7 (7.4)	3.37 (-3.51 to 10.24)	1.84 (0.49 to 6.88)	
Female	8 (3.6)	6 (2.9)	-0.63 (-4.00 to 2.72)	0.82 (0.29 to 2.33)	
**Preoperative oedema**					0.567
<=1cm	8 (3.9)	8 (4.0)	0.02 (-3.77 to 3.81)	1.00 (0.38 to 2.63)	
>1cm	3 (3.1)	5 (5.1)	2.01 (-3.54 to 7.56)	1.65 (0.41 to 6.71)	
**Tumor site**					0.541
Convexity *	6 (4.0)	9 (5.7)	1.70 (-3.10 to 6.40)	1.42 (0.52 to 3.87)	
Others	5 (3.3)	4 (2.8)	-0.50 (-4.50 to 3.50)	0.85 (0.23 to 3.11)	
**TED**					0.942
<=5cm	8 (3.0)	9 (3.4)	0.46 (-2.52 to 0.34)	1.15 (0.45 to 2.95)	
>5cm	3 (10.3)	4 (11.1)	0.80 (-14.30 to 15.90)	1.07 (0.26 to 4.42)	

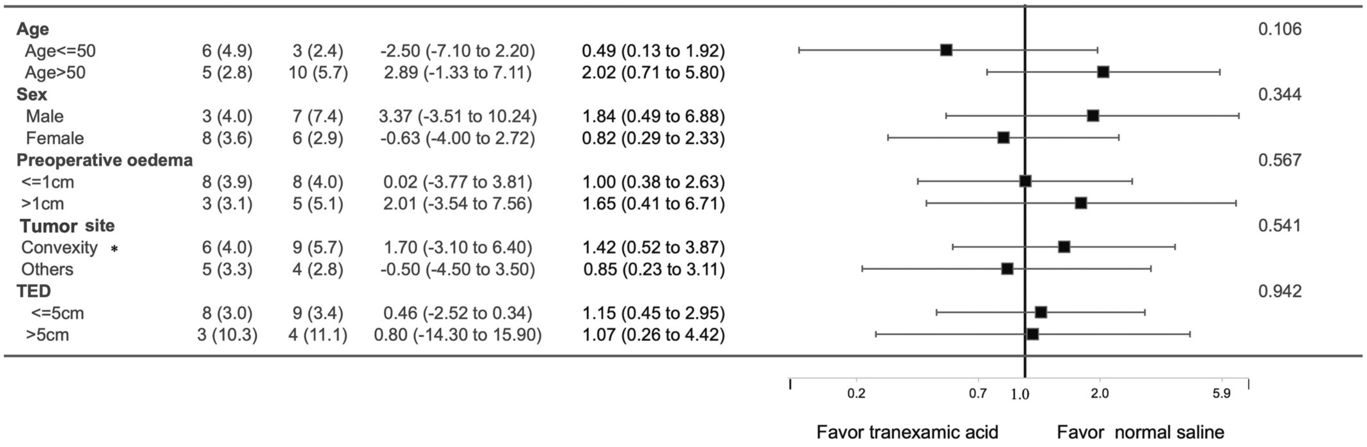

Fig. 2. Risk ratios for early postoperative seizure by subgroup.
There were no significant interactions between treatment and pre-defined subgroups on postoperative delirium.
TED, tumor equivalent diameter.
* Convexity meningioma are tumors that grow on the surface of the brain directly under the skull.

expected after neurosurgery, our incidence was greater than reported in other cardiac and non-cardiac surgery populations, which ranged from 0.2% to 2.7%. [5,7,48]

A single dose of 20 mg/kg TXA was non-inferior to normal saline for postoperative seizures within 7 days. Results were consistent for focal and generalized seizure, and across all pre-defined subgroups and pathology-diagnosed populations. The similar seizure incidence we observed in 600 neurosurgical patients is consistent with a meta-analysis in actively bleeding patients where the risk of seizure was comparable overall but increased somewhat at higher doses. Of note, 83% of included trials were used ≤2 g/day TXA as we did. [35] In contrast, another meta-analysis reports that TXA increases seizure risk 5.4-fold in cardiac surgical patients. [48] But importantly, the lowest TXA dose in any included trial was 24 mg/kg which was slightly higher than the dose we used, and the highest dose was 109 mg/kg. Available data thus suggest that dose matters for seizures, and that no more TXA than necessary should be given. [31]

Our observed mean absolute risk difference for seizures of just 0.7% was well below our noninferiority margin and corresponds to a number-need-to-harm of 143. On average then, TXA 20 mg/kg would need to be given to 143 patients having supratentorial meningioma resections to provoke one additional postoperative seizure. Additionally, there was no evidence that any pre-defined subgroups were at special risk. [36,49–51] Our noninferiority conclusion in a neurological population is consistent with two recent reports indicating that doses of TXA <2 g/per day do not provoke seizures in cardiac and other populations, [7,35] although previous research indicated an increased risk of seizure in cardiac population, [48] possibly related to dose.

The median onset time to postoperative seizure was 2 days which is similar to a previous report in neurosurgical patients, [10] but considerably later than in patients with intracranial hemorrhage population or cardiac surgery in whom seizures generally occur within a day. [6,52] Faster onset time may again be due to higher TXA doses which result in persistent meaningful cerebrospinal fluid concentrations. [17]

Venous thromboembolic events (deep vein thrombosis and pulmonary thrombosis) and ischemic events (myocardial infarction and cerebral ischemia) were similar in each group and of an expected magnitude. [5–7] Furthermore, 7-day complications, length of hospital stay, unplanned ICU admission, length of ICU stay, hospitalization cost, and mechanical ventilation were similar in each group. Overall, our results indicate that TXA is non-inferior to normal saline with respect to postoperative seizures and does not prolong hospital stay or increase thrombotic risk.

The primary purpose of TXA is to reduce bleeding. Nonetheless, we did not observe statistically significant or clinically meaningful differences in blood loss or changes in hemoglobin concentration. Blood loss and the incidence of anemia (hemoglobin <13 g/dL for men and < 12 g/dL for women) was similar in each group, and there were no significant differences in postoperative thrombotic events. TXA thus failed in its primary mission of reducing blood loss, possibly because loss was relatively small.

It seems likely that tumor complexity and surgeons' skill masked the fibrinolytic benefit in our patients. For instance, with similar tumor volume and tumor location, average baseline blood loss in our institute ranged from 300 to 624 mL, [53–55] only a quarter-to-half of that reported in other centers. [22–24] Furthermore, our operations were shorter than usual for such tumors. [24,27] The shorter operation duration and lower baseline blood loss may thus have concealed the drug's blood-sparing effect. A Type-2 statistical error is also possible since blood loss was one of several secondary outcomes and we did not compensate for multiple comparisons. We also note that the TXA dose we used was relatively small to avoid provoking excessive seizures. The TXA dose we selected was a trade-off between bleeding prevention and provoking seizure in our especially vulnerable population. Higher doses would presumably be more effective at reducing blood loss but at the possible cost of additional seizures. [35] TXA, therefore, did not provide benefit in our patients, although it is beyond question that the anti-fibrinolytic agent generally reduces blood loss. [7] What our results add is robust evidence that up to 20 mg/kg of TXA does not provoke seizures, even in patients at extremely high risk.

4.1. Limitation

Due to insufficient evidence supporting the intraoperative prophylactical antiepileptics in preventing postoperative seizure, [40–42] surgeons determined its use according to their clinical preference, and the differences in perioperative prophylactical antiepileptics were negligible. Our trial was restricted to patients having supratentorial meningioma resections. Patients with infratentorial lesions or other intracranial pathologies (subarachnoid hemorrhage, cerebral abscess, and traumatic intracranial hemorrhage) present different risks and mechanisms of seizure or epilepsy. [30] Our findings should therefore be

cautiously extrapolated to other neurosurgical populations. The TXA dose we selected was relatively small. However, selecting the appropriate dose for drug trials is always challenging since investigators must balance benefit and toxicity, which usually increase with dose. TXA 1–2 g has been used in many trials, [3,5–7,56] while the average patient weight of our trial was 66 kg, corresponding to a total of 1.3 g TXA, which is in accordance with previous reports. But in our patients, there was a serious safety concern, namely that TXA provokes seizures — and patients having extensive brain surgery are presumably at special risk. We thus compromised on a dose that was likely to be both effective and safe. An additional limitation is that seizures were largely identified clinically not electroencephalogram-diagnosed, as is usual for TXA studies, [5,7] and restricted to the initial 7 postoperative days. We therefore surely under-estimated subclinical seizures. However, the incidence of postoperative seizure was similar to previous reports, [8] though lower than anticipated and leading to the possibility of underpower, the conclusion of non-inferiority is reasonable and reliable. And finally, our trial was restricted to a single-center, high-volume center, and all patients were Chinese.

5. Conclusion

In summary, among patients having supratentorial meningioma resection, a single intraoperative dose of TXA did not significantly reduce bleeding and was non-inferior with respect to postoperative seizures within 7 days after surgery.

Supplementary data to this article can be found online at https://doi.org/10.1016/j.jclinane.2023.111285.

Disclosure

This work was supported by the Ministry of Science and Technology of the People's Republic of China funding (2018YFC2001901), Beijing Municipal Science & Technology Commission (Z191100006619068) and Beijing Municipal Administration of Hospitals Incubating Program (PX2022018).

Data sharing statement

See Supplementary File 2.

CRediT authorship contribution statement

Shu Li: Methodology, Formal analysis, Writing – original draft, Funding acquisition. **Minying Liu:** Investigation, Data curation. **Jingchao Yang:** Investigation. **Xiang Yan:** Conceptualization, Investigation. **Yaru Wu:** Investigation. **Liyong Zhang:** Investigation. **Min Zeng:** Investigation. **Dabiao Zhou:** Resources. **Yuming Peng:** Conceptualization, Resources, Writing – original draft, Supervision, Funding acquisition. **Daniel I. Sessler:** Writing – review & editing.

Declaration of Competing Interest

The authors declare no competing interests.

Acknowledgment

We deeply thank Dr. Song Lin (Department of Neurosurgery, Beijing Tiantan Hospital, Capital Medical University, Beijing, China) for his contributions and dedication to the neurosurgical management of participants; compensation was not received.

References

[1] Zhao Y, Xi C, Xu W, Yan J. Role of tranexamic acid in blood loss control and blood transfusion management of patients undergoing multilevel spine surgery: a meta-analysis. Medicine (Baltimore) 2021;100:e24678.
[2] Nikolaou VA-O, Masouros P, Floros T, Chronopoulos E, Skertsou M, Babis GA-O. Single dose of tranexamic acid effectively reduces blood loss and transfusion rates in elderly patients undergoing surgery for hip fracture: a randomized controlled trial. Bone Joint J 2021;103-8:442–8.
[3] Heyns M, Knight P, Steve AK, Yeung JK. A single preoperative dose of tranexamic acid reduces perioperative blood loss: a meta-analysis. Ann Surg 2021;273:75–81.
[4] Almuwallad A, Cole E, Ross J, Perkins Z, Davenport R. The impact of prehospital TXA on mortality among bleeding trauma patients: a systematic review and meta-analysis. J Trauma Acute Care Surg 2021;90:901–7.
[5] Shi J, Zhou C, Pan W, Sun H, Liu S, Feng W, et al. Effect of high- vs low-dose tranexamic acid infusion on need for red blood cell transfusion and adverse events in patients undergoing cardiac surgery: the OPTIMAL randomized clinical trial. Jama 2022;328:336–47.
[6] Sprigg N, Flaherty K, Appleton JP, Al-Shahi Salman R, Bereczki D, Beridze M, et al. Tranexamic acid for hyperacute primary intracerebral haemorrhage (TICH-2): an international randomised, placebo-controlled, phase 3 superiority trial. Lancet 2018;391:2107–15.
[7] Devereaux PJ, Marcucci M, Painter TW, Conen D, Lomivorotov V, Sessler DI, et al. Tranexamic acid in patients undergoing noncardiac surgery. N Engl J Med 2022;386:1986–97.
[8] Li X, Wang C, Lin Z, Zhao M, Ren X, Zhang X, et al. Risk factors and control of seizures in 778 Chinese patients undergoing initial resection of supratentorial meningiomas. Neurosurg Rev 2020;43:597–608.
[9] Wirsching HG, Morel C, Gmur C, Neidert MC, Baumann CR, Valavanis A, et al. Predicting outcome of epilepsy after meningioma resection. Neuro Oncol 2016;18:1002–10.
[10] Ersoy TF, Ridwan S, Grote A, Coras R, Simon M. Early postoperative seizures (EPS) in patients undergoing brain tumour surgery. Sci Rep 2020;10:13674.
[11] Horiuchi S, Kanaya K, Horiuchi T. The occurrence and relationship of postoperative seizure and de novo epilepsy after craniotomy surgery: a retrospective single-center cohort study. Front Surg 2022;9:881874.
[12] Furtmüller R, Schlag MG, Berger M, Hopf R, Huck S, Sieghart W, et al. Tranexamic acid, a widely used antifibrinolytic agent, causes convulsions by a gamma-aminobutyric acid(A) receptor antagonistic effect. J Pharmacol Exp Ther 2002;301:168–73.
[13] Lecker I, Wang DS, Romaschin AD, Peterson M, Mazer CD, Orser BA. Tranexamic acid concentrations associated with human seizures inhibit glycine receptors. J Clin Invest 2012;122:4654–66.
[14] Merino JG, Latour LL, Tso A, Lee KY, Kang DW, Davis LA, et al. Blood-brain barrier disruption after cardiac surgery. AJNR Am J Neuroradiol 2013;34:518–23.
[15] Stone TJ, Rowell R, Jayasekera BAP, Cunningham MO, Jacques TS. Review: molecular characteristics of long-term epilepsy-associated tumours (LEATs) and mechanisms for tumour-related epilepsy (TRE). Neuropathol Appl Neurobiol 2018;44:56–69.
[16] Adhikari S, Walker BC, Mittal S. Pathogenesis and management of brain tumor-related epilepsy. In: Debinski W, editor. Gliomas. Brisbane (AU): Exon publications copyright: the authors; 2021.
[17] Lecker I, Wang DS, Whissell PD, Avramescu S, Mazer CD, Orser BA. Tranexamic acid-associated seizures: causes and treatment. Ann Neurol 2016;79:18–26.
[18] de Faria JL, da Silva Brito J, Costa ESLT, Kilesse C, de Souza NB, Pereira CU, et al. Tranexamic acid in neurosurgery: a controversy indication-review. Neurosurg Rev 2021;44:1287–98.
[19] Effects of tranexamic acid on death, disability, vascular occlusive events and other morbidities in patients with acute traumatic brain injury (CRASH-3): a randomised, placebo-controlled trial. Lancet 2019;394:1713–23.
[20] Lotan R, Lengenova S, Rijini N, Hershkovich O. Intravenous tranexamic acid reduces blood loss in multilevel spine surgeries. J Am Acad Orthop Surg 2022;31:e226–30.
[21] Paulo D, Semonche A, Choudhry O, Al-Mufti F, Prestigiacomo CJ, Roychowdhury S, et al. History of hemostasis in neurosurgery. World Neurosurg 2019;124:237–50.
[22] Ravi GK, Panda N, Ahluwalia J, Chauhan R, Singla N, Mahajan S. Effect of tranexamic acid on blood loss, coagulation profile, and quality of surgical field in intracranial meningioma resection: a prospective randomized, double-blind, placebo-controlled study. Surg Neurol Int 2021;12:272.
[23] Rebai L, Mahfoudhi N, Fitouhi N, Daghmouri MA, Bahri K. Intraoperative tranexamic acid use in patients undergoing excision of intracranial meningioma: randomized, placebo-controlled trial. Surg Neurol Int 2021;12:289.
[24] Hooda B, Chouhan RS, Rath GP, Bithal PK, Suri A, Lamsal R. Effect of tranexamic acid on intraoperative blood loss and transfusion requirements in patients undergoing excision of intracranial meningioma. J Clin Neurosci 2017;41:132–8.
[25] Li S, Yan X, Li R, Zhang X, Ma T, Zeng M, et al. Safety of intravenous tranexamic acid in patients undergoing supratentorial meningiomas resection: protocol for a randomised, parallel-group, placebo control, non-inferiority trial. BMJ Open 2022;12:e052095.
[26] Stevens PE, Levin A. Evaluation and management of chronic kidney disease: synopsis of the kidney disease: improving global outcomes 2012 clinical practice guideline. Ann Intern Med 2013;158:825–30.
[27] Mebel D, Akagami R, Flexman AM. Use of tranexamic acid is associated with reduced blood product transfusion in complex skull base neurosurgical procedures: a retrospective cohort study. Anesth Analg 2016;122:503–8.

[28] Vel R, Udupi BP, Satya Prakash MV, Adinarayanan S, Mishra S, Babu L. Effect of low dose tranexamic acid on intra-operative blood loss in neurosurgical patients. Saudi J Anaesth 2015;9:42–8.

[29] Sprigg N, Flaherty K, Appleton JP, Al-Shahi Salman R, Bereczki D, Beridze M, et al. Tranexamic acid to improve functional status in adults with spontaneous intracerebral haemorrhage: the TICH-2 RCT. Health Technol Assess 2019;23:1–48.

[30] Dadure C, Sauter M, Bringuier S, Bigorre M, Raux O, Rochette A, et al. Intraoperative tranexamic acid reduces blood transfusion in children undergoing craniosynostosis surgery: a randomized double-blind study. Anesthesiology 2011;114:856–61.

[31] Picetti R, Shakur-Still H, Medcalf RL, Standing JF, Roberts I. What concentration of tranexamic acid is needed to inhibit fibrinolysis? A systematic review of pharmacodynamics studies. Blood Coagul Fibrinolysis 2019;30:1–10.

[32] Boysen SR, Pang JM, Mikler JR, Knight CG, Semple HA, Caulkett NA. Comparison of tranexamic acid plasma concentrations when administered via intraosseous and intravenous routes. Am J Emerg Med 2017;35:227–33.

[33] Grassin-Delyle S, Semeraro M, Foissac F, Bouazza N, Shakur-Still H, Roberts I, et al. Tranexamic acid through intravenous, intramuscular and oral routes: an individual participant data meta-analysis of pharmacokinetic studies in healthy volunteers. Fundam Clin Pharmacol 2019;33:670–8.

[34] Grassin-Delyle S, Shakur-Still H, Picetti R, Frimley L, Jarman H, Davenport R, et al. Pharmacokinetics of intramuscular tranexamic acid in bleeding trauma patients: a clinical trial. Br J Anaesth 2021;126:201–9.

[35] Murao S, Nakata H, Roberts I, Yamakawa K. Effect of tranexamic acid on thrombotic events and seizures in bleeding patients: a systematic review and meta-analysis. Crit Care 2021;25:380.

[36] Chen WC, Magill ST, Englot DJ, Baal JD, Wagle S, Rick JW, et al. Factors associated with pre- and postoperative seizures in 1033 patients undergoing supratentorial meningioma resection. Neurosurgery 2017;81:297–306.

[37] National Clinical Guideline C. National institute for health and care excellence: guidelines. blood transfusion. London: National Institute for Health and Care Excellence (NICE) Copyright © 2015 National Clinical Guideline Centre; 2015.

[38] Klein AA, Bailey CR, Charlton AJ, Evans E, Guckian-Fisher M, McCrossan R, et al. Association of Anaesthetists guidelines: cell salvage for peri-operative blood conservation 2018. Anaesthesia 2018;73:1141–50.

[39] Liang S, Fan X, Chen F, Liu Y, Qiu B, Zhang K, et al. Chinese guideline on the application of anti-seizure medications in the perioperative period of supratentorial craniocerebral surgery. Ther Adv Neurol Disord 2022;15. 17562864221114357.

[40] Greenhalgh J, Weston J, Dundar Y, Nevitt SJ, Marson AG. Antiepileptic drugs as prophylaxis for postcraniotomy seizures. Cochrane Database Syst Rev 2020;4. Cd007286.

[41] Walbert T, Harrison RA, Schiff D, Avila EK, Chen M, Kandula P, et al. SNO and EANO practice guideline update: anticonvulsant prophylaxis in patients with newly diagnosed brain tumors. Neuro Oncol 2021;23:1835–44.

[42] Chandra V, Rock AK, Opalak C, Stary JM, Sima AP, Carr M, et al. A systematic review of perioperative seizure prophylaxis during brain tumor resection: the case for a multicenter randomized clinical trial. Neurosurg Focus 2017;43:E18.

[43] Fisher RS, Cross JH, D'Souza C, French JA, Haut SR, Higurashi N, et al. Instruction manual for the ILAE 2017 operational classification of seizure types. Epilepsia 2017;58:531–42.

[44] Jaramillo S, Montane-Muntane M, Gambus PL, Capitan D, Navarro-Ripoll R, Blasi A. Perioperative blood loss: estimation of blood volume loss or haemoglobin mass loss? Blood Transfus 2020;18:20–9.

[45] Mueller MM, Van Remoortel H, Meybohm P, Aranko K, Aubron C, Burger R, et al. Patient blood management: recommendations from the 2018 Frankfurt consensus conference. JAMA 2019;321:983–97.

[46] Lopez-Picado A, Albinarrate A, Barrachina B. Determination of perioperative blood loss: accuracy or approximation? Anesth Analg 2017;125:280–6.

[47] Jaramillo S, Montane-Muntane M, Capitan D, Aguilar F, Vilaseca A, Blasi A, et al. Agreement of surgical blood loss estimation methods. Transfusion 2019;59:508–15.

[48] Lin Z, Xiaoyi Z. Tranexamic acid-associated seizures: a meta-analysis. Seizure 2016;36:70–3.

[49] Elbadry Ahmed R, Tang H, Asemota A, Huang L, Boling W, Bannout F. Meningioma related epilepsy- pathophysiology, pre/postoperative seizures predicators and treatment. Front Oncol 2022;12:905976.

[50] Hwang K, Joo JD, Kim YH, Han JH, Oh CW, Yun CH, et al. Risk factors for preoperative and late postoperative seizures in primary supratentorial meningiomas. Clin Neurol Neurosurg 2019;180:34–9.

[51] Schneider M, Güresir Á, Borger V, Hamed M, Rácz A, Vatter H, et al. Preoperative tumor-associated epilepsy in patients with supratentorial meningioma: factors influencing seizure outcome after meningioma surgery. J Neurosurg 2019:1–7.

[52] Manji RA, Grocott HP, Leake J, Ariano RE, Manji JS, Menkis AH, et al. Seizures following cardiac surgery: the impact of tranexamic acid and other risk factors. Can J Anaesth 2012;59:6–13.

[53] Wang B, Zhang GJ, Wu Z, Zhang JT, Liu PN. Surgical outcomes and prognostic factors of parasagittal meningioma: a single-center experience 165 consecutive cases. Br J Neurosurg 2021:1–7.

[54] Lin Z, Zhao M, Li X, Wang J, Qiu P, Lan F, et al. Characteristic features and proposed classification in 69 cases of intracranial microcystic meningiomas. Neurosurg Rev 2019;42:443–53.

[55] Ma J, Cheng L, Wang G, Lin S. Surgical management of meningioma of the trigone area of the lateral ventricle. World Neurosurg 2014;82:757–69.

[56] Nikolaou VS, Masouros P, Floros T, Chronopoulos E, Skertsou M, Babis GC. Single dose of tranexamic acid effectively reduces blood loss and transfusion rates in elderly patients undergoing surgery for hip fracture: a randomized controlled trial. Bone Joint J 2021;103-B:442–8.
