# Supplementary material for: Efficacy and safety of tranexamic acid on blood loss and seizures in patients undergoing meningioma resection: A systematic review and meta-analysis
Source: PLoS One. 2024 Sep 4;19(9):e0308070. doi: 10.1371/journal.pone.0308070 (PMC11373793; doi:10.1371/journal.pone.0308070)
Supplement: Attachment — Submitted filename: Response to reviewers_0701.docx [file pone.0308070.s005.docx]

**Response to Reviewers:**

**Reviewer #1:**

The search strategy is not clear/available. Literature suggests that Tranexamic acid can precipitate seizures in certain doses and there is controversy regarding the role of prevention of seizures. It is not clear whether the patients in either group were on anti-epileptics. Study needs to more elaborative.

**Response:** Thanks. The search strategy for this paper was: ((tranexamic acid) OR (TXA) OR (Tranexamic acid)) AND (meningioma) AND ((randomized controlled trial) OR (randomized)). Tranexamic acid can precipitate seizures in certain doses, and we found that tranexamic acid did not increase the incidence of seizure in our meta-analysis. Additionally, the meta-analysis included four studies, of which only one mentioned the prophylactic administration of antiepileptic drugs postoperatively, while the remaining three did not specify whether antiepileptic treatment was given.

| Author, year | Whether to use postoperative antiepileptic drugs administration |
| --- | --- |
| Hooda et al., 2017 | None. |
| Rebai et al., 2021 | None. |
| Ravi et al., 2021 | None. |
| Shu Li et al., 2023 | Yes. Postoperative antiepileptic treatment includes intravenous or oral administration of valproic acid, oxcarbazepine, carbamazepine, levetiracetam, and phenobarbital. Rescue intramuscular phenobarbital or continuous infusion of midazolam was administered when seizure fit attacks. |

**Related revised manuscript text:** Only one study mentioned the prophylactic administration of antiepileptic drugs postoperatively, while the other studies did not specify this.

**Manuscript location:** Page 9, line 19- and table 1 last column in the revised manuscript.

**Reviewer #2:**

I find it a well-designed paper. This study demonstrates that the administration of TXA could reduce blood loss (by 252 mL) in patients undergoing intracerebral meningioma resection. Meanwhile, TXA did not increase the risk of seizure, or other postoperative complications. I, however, have a few minor comments to make:

**Comment #1:** Meningiomas are one of the most common intracranial tumors. Line 232, on page 20, with null or hard-to-interpret outcomes may not be published; please, the author explicitly states the meaning of “hard-to-interpret outcomes.”

**Response:** Thanks. We acknowledge that the term “hard-to-interpret” is inappropriate and have removed it.

**Related revised manuscript text:** In addition, we collected studies through systematically searching selected databases, nevertheless the potential for publication bias persists.

**Manuscript location:** Page 12, line 14- in the revised manuscript.

**Comment #2:**

Line 243-245, on page 21, This systematic review and meta-analysis suggests that the administration of TXA could reduce blood loss (by 252 mL) in patients undergoing intracerebral meningioma resection. The detailed blood loss (by 252) is inappropriate in the conclusion section.

**Response:** Thanks. We agree with you and have moved the data in the conclusion section.

**Related revised manuscript text:** This systematic review and meta-analysis suggests that the administration of TXA could reduce blood loss in patients undergoing intracerebral meningioma resection.

**Manuscript location:** Page 13, line 5- in the revised manuscript.

**Reviewer #3:**

Thank you for this valuable research. The manuscript is presented in a very good, informative and organised way. It is also written in a standard English language. Only the differences between the studies you choose for your analysis are multiple.

**Response:** Thanks. We appreciate your positive comments on the manuscript. We acknowledge that the multiple analysis in the studies chose in the meta which also led to the high value of *I^2^*. We also discussed this in detail in the limitations section to clarify the impact of these factors on our results.

**Related revised manuscript text:** The multiple analyses in the studies chose in the meta also led to the high value of *I^2^*.

**Manuscript location:** Page 12, line 17- in the revised manuscript.

**Reviewer #4:**

In their response letter, authors claim that recent study showed that TMZ did not add any benefit in the treatment of IDH wildtype glioblastoma compared with radiotherapy alone, regardless of MGMT promoter status (https://pubmed.ncbi.nlm.nih.gov/34000245/. This is simply a misinterpretation: Adjuvant temozolomide chemotherapy, but not concurrent temozolomide chemotherapy, was associated with a survival benefit in patients with 1p/19q non-co-deleted anaplastic gliomas. I suggest to change the passages in Discussion accordingly. I suggest to re-write the conclusion and state that the effects of the two drugs were shown in an in-vitro animal model under laboratory circumstances, and that it by no means suggest a positive clinical perspective for patients with necessity of further studies.

**Response:** Thanks. The reviewer’s comment appears to not correspond to our paper.
