# Supplementary material for: Efficacy and safety of tranexamic acid on blood loss and seizures in patients undergoing meningioma resection: A systematic review and meta-analysis
Source: PLoS One. 2024 Sep 4;19(9):e0308070. doi: 10.1371/journal.pone.0308070 (PMC11373793; doi:10.1371/journal.pone.0308070)
Supplement: Attachment — Submitted filename: LS-reviewer comments-0711.docx [file pone.0308070.s006.docx]

**Response to Reviewers:**

**Reviewer #4:** Authors present a revised version of their manuscript -systematic review on role of transexamic acid in resection of meningiomas for prevention of postoperative bleeding as well as on prevention of seizures, where following anaylsis of four cohorts and more than 700 patients their main conclusion was that TXA reduces blood loss without increasing the risk of seizures. All limitations which were adressed, such as the fact that 3/4 cohort studies did not report use of anticonvulsive therapy - this needs to be adressed in the Conclusions and in Discussion.

**Response:** Thanks. We accepted your suggestion and added the limitation in the part of the conclusion and discussion.

**Related revised manuscript text:**

Last, though two of the four studies excluded patients with preoperative seizures, only one study reported the use of anticonvulsive therapy, leading to an inconclusive effect of TXA on postoperative seizure.

However, due to the heterogeneity of studies, the impact of TXA on the risk of seizure or other postoperative complications remains inconclusive. Large multi-center clinical trials are needed to fully confirm the effect of TXA administration in saving blood loss and postoperative seizures in the neurosurgical population.

**Manuscript location:** Page 13, line 6-8, line 13-16- in the revised manuscript.
